# Bridging gaps in breast cancer care: a Breast Cancer Care Quality Index to improve outcomes worldwide

**DOI:** 10.3332/ecancer.2025.1981

**Published:** 2025-09-01

**Authors:** Eduardo Cazap, Benjamin O Anderson, Giuseppe Curigliano, Sandeep Sehdev, Fatima Cardoso, Ana Rita Gonzalez, Emad Shash, Cheng-Har Yip, André Mattar, Yanin Chavarri-Guerra, Miriam Mutebi, Yongmei Yin, João Victor Rocha, Ilaria Lucibello, Namita Srivastava

**Affiliations:** 1 Latin-American and Caribbean Society of Medical Oncology (SLACOM), Buenos Aires CP 1057, Argentina; 2 University of Washington, Seattle, WA 98195, USA; 3 Department of Oncology and Hemato-Oncology, University of Milano, Milano 20122, Italy; 4 European Institute of Oncology, IRCCS, Milano 20141, Italy; 5 Ottawa Hospital Cancer Centre, Ottawa, ON K1H 1C4, Canada; 6 Advanced Breast Cancer (ABC) Global Alliance, Lisbon 1400-038, Portugal; 7 Policy Wisdom LLC, Quebradillas 00678-2705, Puerto Rico; 8 Medical Oncology, National Cancer Institute, Cairo University, Giza 12613, Egypt; 9 Picaso Hospital, Petaling Jaya 46300, Malaysia; 10 University of Malaya, Kuala Lumpur 50603, Malaysia; 11 Mastology Department, Women’s Health Hospital, São Paulo 01215-000, Brazil; 12 Instituto Nacional de Ciencias Médicas y Nutrición Salvador Zubirán, Mexico City 40080, Mexico; 13 Department of Surgery, Aga Khan University, Nairobi 30270-00100, Kenya; 14 Department of Oncology, The First Affiliated Hospital of Nanjing Medical University, Nanjing 210000–211300, China; 15 State Key Laboratory Cultivation Base of Biomarkers for Cancer Precision Prevention and Treatment, Collaborative Innovation Center for Cancer Personalized Medicine, Nanjing Medical University, Nanjing 210029, China; # These authors contributed equally to this work.; + These authors contributed equally to this work.

**Keywords:** breast cancer, quality index, early detection, timely diagnosis, comprehensive management, healthcare systems strengthening, health equity, patient centricity, universal access to health, healthcare quality, treatment effectiveness

## Abstract

**Background:**

Breast cancer (BC) care faces challenges in early detection, timely diagnosis and comprehensive management. Disparities persist, with underserved populations facing the greatest barriers. Addressing these requires policies that support consistent, evidence-based practices and enhance healthcare capacity and technology advancements. This document presents the development of the Breast Cancer Care Quality Index (BCCQI), supported by evidence to promote equitable care and improve BC outcomes globally, and discusses its adoption as a strategic tool within National Cancer Control Plans.

**Methods:**

A two-part methodology identified challenges in BC care and defined dimensions, targets and indicators for the BCCQI, aligned with the World Health Organization Global Breast Cancer Initiative. A literature review and analysis of existing United Nations (UN) frameworks informed the initial structure of the index, which was later refined through expert feedback from a multidisciplinary panel representing diverse backgrounds and geographies.

**Findings:**

The BCCQI is organised into four dimensions, comprising 10 targets and 23 indicators to guide the development of country-specific roadmaps. It should promote progress across key domains: health equity, patient centricity, universal access, care quality and treatment effectiveness. The Index is conceived as a dynamic tool, continuously refined through real-world application and emerging evidence.

**Interpretation:**

Despite the previous initiatives, progress has been slow, likely due to practical details and country-specific guidance remaining limited due to scarce real-world evidence. Promoting national ownership and empowering action aligned with local challenges and opportunities, a flexible, strategic framework may help address these gaps.

## Introduction

Breast cancer (BC) was the most prevalent cancer in 2022, affecting over 8.1 million individuals worldwide [1]. It has a profoundly debilitating impact on patients and imposes substantial individual, societal and economic burdens. Despite significant advancements in BC research, diagnosis and treatment, substantial challenges persist. Access to optimal care remains limited, particularly in low- and middle-income countries (LMICs) [2]. Moreover, integrated, patient-centered approaches are not broadly available, with underserved populations facing the greatest barriers. These disparities highlight that challenges in BC care endure globally, leading to inequities both between and within countries [3–5]. 

Over time, significant efforts have been undertaken to guide and support countries in strengthening their national BC programs and service provision to improve patient outcomes, like for example, the Breast Health Global Initiative (BHGI) [6, 7] and the BC Initiative 2.5 (BCI2.5) [8, 9]. More recently, to achieve a 2.5% annual decrease in BC mortality rates at the global level, the World Health Organization (WHO) Global Breast Cancer Initiative (GBCI) provided *evidence-based recommendations for countries to follow a phased approach to implementing interventions focused on improving BC early detection, diagnosis and treatment*. The GBCI, which also describes the BC patient journey, identifies three evidence-based key performance indicators (KPIs) to pinpoint country-level gaps [2].

However, recent data indicate that only a few countries have achieved or are on track to achieve-the GBCI goal [5]. This suggests that further support might be needed to help countries more effectively and rapidly scale up their efforts to improve BC care.

A comprehensive approach aimed at reducing disparities and promoting equitable care for all individuals will require bold policy change in support of consistent, evidence-based practices and a two-pronged approach focused on increasing the existing healthcare system capacity while aligning rapid advancements in health technologies. Such an undertaking calls for expanding action on BC care and engaging a more diverse and comprehensive group of stakeholders to drive collaboration and shared responsibility.

To increase clarity around the required commitment and accountability for improving the quality of BC care and outcomes for all patients, we aspire for a new Breast Cancer Care Quality Index (BCCQI) to be integrated into National Cancer Control Plans (NCCPs). Building on the GBCI, the BCCQI will provide additional guidance on essential elements to enhance national-level BC care quality. The guidance provided should allow countries to tailor their own implementation plans to their specific needs and develop context-sensitive roadmaps to adapt over time as progress is achieved. This flexible and iterative approach encourages national ownership, supports inclusive participation and should facilitate incremental progress across five key domains essential to BC care improvement: health equity, patient centricity, universal access to health, healthcare quality and treatment effectiveness.

The objective of this document is to discuss the development of the BCCQI and facilitate alignment on its adoption and implementation as a strategic tool to advance BC care globally.

## Methods

A two-part methodology was developed, adapted from the consensus approach [10], in which we aimed to gather general agreement on the key components to be included in the BCCQI, in order to address the limitations of empirical data supporting the impact and expected outcomes of commonly used metrics across real-world settings.

### 1. Parallel reviews to inform the preliminary framework

#### Peer-reviewed literature

A review of global evidence explored the multidimensional impact of BC, and the challenges and priority areas for quality improvement across the BC care continuum. We used Medical Subject Headings search terms on PubMed, including ‘breast cancer’, ‘breast cancer detection’, ‘breast cancer diagnosis’ and ‘breast cancer treatment’. The search was conducted between April 14th and May 24th, 2024. Target papers were identified based on their relevance to BC burden and challenges. Relevance to the BC burden was assessed based on evidence related to: (i) the disease burden, including BC incidence, mortality and disability-adjusted life years; (ii) the socio-economic impact, encompassing direct healthcare costs, out-of-pocket expenditures and productivity losses and (iii) the impact on the well-being of individuals and caregivers, including effects on physical and mental health, as well as on personal and professional relationships. Relevance to BC challenges was evaluated based on the extent to which the evidence addressed barriers encountered across the patient journey – from early detection and diagnosis to treatment, survivorship and recovery. A total of 89 papers were considered in the preliminary literature review.

#### Grey literature

Policy documents from governmental and international sources were reviewed to assess existing BC strategies, guidelines and recommendations and to identify potential areas for policy improvement.

#### United Nations (UN) resolutions and frameworks

Relevant UN health resolutions and frameworks were reviewed to shape the BCCQI’s structure (Box 1).

Based on the analysis of the literature, the BCCQI was aligned with the three pillars of the GBCI framework – health promotion, timely diagnosis and comprehensive treatment – as well as the patient pathway outlined within the GBCI.

A fourth dimension related to healthcare systems resilience was included as supported by literature review findings.

The parallel reviews provided valuable insights into the key issues the BCCQI should address, potential indicators for assessment, areas for policy improvement and the approach used by existing BC strategies. These findings informed the development of the preliminary BCCQI framework, with evidence justifying the inclusion of each target and indicator, which was subsequently refined through expert feedback.

### 2. Expert feedback

A multi-disciplinary group of 18 experts was consulted to provide key insights and feedback and to endorse the BCCQI through a three-phase feedback process. These experts were selected to ensure a comprehensive perspective on BC care, and with consideration for:

1. Affiliations and roles within reputable organisations dedicated to BC.
2. Diverse range of geographic locations.
3. Active engagement and participation in BC policymaking and guideline development.
4. Active involvement in patient advocacy through international and regional organisations dedicated to BC and its care.
5. Relevant publications and evidence of familiarity with the latest advancements in BC care.

Box 1. **Example of UN-related frameworks reviewed to inform the Breast Cancer Care Quality Index (BCCQI).**

- The 2030 Agenda for Sustainable Development
- The WHO Noncommunicable Disease (NCD) Global Monitoring Framework
- Monitoring and Evaluation of the Global Antimicrobial Resistance (AMR) Action Plan
- Indicator and Monitoring Framework for the Global Strategy for Women’s, Children’s and Adolescents’ Health
- Monitoring Framework for Universal Health in the Americas

The group comprised 11 experts selected for their experience as healthcare professionals, four as policymakers and public health specialists and three as patient and civil society representatives. It also included eight experts in medical and clinical oncology, with a strong focus on BC and four experts in breast surgery. A wide range of nationalities was represented, covering different regions. These included: four experts from the United States of America, two from Canada and one expert from each of the following countries: Argentina, Brazil, China, Egypt, Italy, Kenya, Malaysia, Mexico, Nigeria, Portugal, Puerto Rico and the United Arab Emirates. The expert feedback and validation process included:

- Multistep expert preliminary feedback (May–July 2024)

This phase included a variety of engagement formats, including in-person and virtual advisory boards, as well as one-on-one meetings. During these sessions, experts discussed key challenges in BC care that the BCCQI should address and reviewed and endorsed the main structure of the preliminary BCCQI.

Following this round of discussions, a more refined second iteration of the Index was developed, supported by evidence justifying the inclusion of each target and indicator.

- Pre-panel survey (October 2024)

In this pre-panel survey, experts were presented with the refined version of the BCCQI. For each indicator, they were asked to either validate it, validate it with modifications or indicate that it should not be validated. Additionally, they were encouraged to provide supporting evidence and sources as needed, addressing aspects such as the clinical and medical accuracy of the topics and the feasibility of data collection.

Following the pre-panel survey, the Index was refined and a third version was developed and supported by relevant literature.

- Two expert panels (January 2025)

The panels were held to address outstanding issues in the near-final version of the BCCQI, in which experts could provide input and suggestions for further refinement. During these sessions, all outstanding issues achieved a minimum of 80% alignment among participants.

Following the two expert panels, the final version of the BCCQI was reached.

- Manuscript development, validation and finalisation

The manuscript was developed concurrently with the expert validation process, summarising key findings from the literature review and the expert feedback process. Twelve experts who contributed to the full process for the development of the BCCQI are listed as co-authors of the manuscript.

## Background and context

### Breast cancer: biological, clinical, epidemiological and socioeconomic perspectives

Breast cancer is the most common cancer among women in most countries and represents an important cause of premature mortality globally [2]. It imposes a staggering burden, straining healthcare systems, impacting the quality of life for patients and their families and with a great socioeconomic cost. The International Agency for Research on Cancer (IARC) estimates nearly 2.3 million new BC cases and 700,000 deaths globally in 2022 [1, 11], with 10.7% of new cases occurring in people aged less than 40 years [12]. In the absence of bold action, the burden of BC is projected to increase to 3.19 million cases by 2040, with around 1.04 million deaths per year [11]. Globally, it has been estimated that BC had an economic cost of 1.964 trillion international dollars in 2017 [13]. Beyond the economic strain and impact on productivity, BC itself and its treatment profoundly affect patients' psychosocial well-being and physical health. BC patients have an increased risk of mental health conditions such as depression and anxiety compared to individuals without BC [14, 15]. In addition, BC is associated with social stigma, along with self-stigma and associative stigma affecting family members, due to the value placed on women’s reproductive capacity or marital status [16]

The causes of BC are poorly understood, and most identified risk factors are not amenable to change [17]; therefore, improvements in early detection, timely diagnosis and comprehensive management are crucial to reducing mortality rates, enhancing patient quality of life and alleviating the economic and social burdens associated with this disease [2]. 

A recent study highlighted that countries with the highest human development index (HDI) show the highest age-adjusted BC incidence rates but the lowest BC mortality rates. Conversely, the countries with lower HDI scores have the lowest incidence but the highest BC mortality rates [5]. In addition, projections indicate that, between 2022 and 2050, BC incidence and mortality will increase more sharply in regions with limited resources [5].

The disproportionately greater mortality faced by LMICs might be affected by country-specific factors, like population structure, lifestyle, genetic factors and environment [18, 19].

Certain groups of women may face higher risks of developing BC, or of developing it at a younger age or experience more aggressive forms of the disease. For example, Ashkenazi Jewish women are at a significantly higher risk of developing BC than other Caucasian women [20, 21]. Within this group, a family history of BC further amplifies the risk, especially for early-onset disease. Similarly, African, Asian and Hispanic/Latina women are more likely to be diagnosed with BC at younger ages compared to their White or Western counterparts [19, 22–26].

However, access to and quality of BC detection, diagnosis and treatment services [27, 28] are also significant drivers of the inequities observed, as shown for example by the striking difference in BC stage at diagnosis, with only about 5%–10% of patients with metastatic BC at initial diagnosis in high-income countries (HICs), compared to up to 50%–80% of patients diagnosed with de novo stage III/IV BC, in LMICs [16, 29–31].

### Breast cancer patient journey

**Figure 1.**
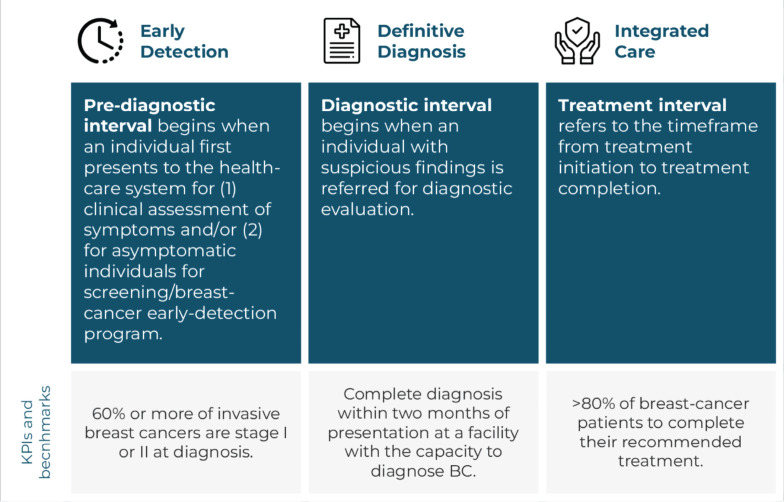
Overview of the three pillars of the GBCI BC patient journey.

The GBCI framework outlines the BC patient journey through three pillars, each corresponding to sequential patient-care intervals, as illustrated in Figure 1. For each pillar, the WHO provides a detailed description of the relevant interval, including the associated processes, interventions and the desired outcomes, which serve as benchmarks for countries to aspire to in improving BC care [2].

### Challenges in BC care: findings from the literature review

Numerous challenges affect the quality of the BC care delivered at the country level. These challenges are often particularly pronounced in LMICs and among vulnerable populations that are more significantly impacted by access barriers, leading to worse patient outcomes [16, 29, 30, 32]. Table 1 presents key challenges identified along the BC patient journey in the initial literature review.

Based on the challenges described above, our analysis led to the identification of the following four dimensions: early detection, timely diagnosis, comprehensive management and strong and resilient healthcare systems.

Early detection and timely diagnosis are vital for reducing BC mortality; analysis of evidence from 1990 to 2020 showed that only countries detecting at least 60% of the invasive cancers at stages I or II achieved a 2% reduction in BC mortality for three consecutive years [2]. Timely diagnosed BCs are treated more effectively, with treatment being better tolerated and less costly [2]. Comprehensive care combines timely cancer therapies (surgery, radiotherapy and systemic treatments) and supportive services, following WHO guidelines and international standards, to ensure holistic BC management, improve patient outcomes and enhance quality of life. Strong, resilient healthcare systems are essential to meet the growing BC burden while ensuring quality, equity and patient-centered care across the BC patient journey [53, 62, 63].

## Results

### Importance of a BC care quality index

Breast cancer is a public health priority for health systems and countries worldwide, given its significant impact on patients, families, health systems and society overall. However, countries are tasked with the challenging responsibility of addressing patients’ healthcare needs while ensuring the economic sustainability of their healthcare system [64, 65]. Considering the projected growing burden of BC, this task appears even more daunting.

To support these efforts, a multitude of initiatives and organisations have focused over the years on providing guidance for countries, healthcare systems and individual healthcare institutions, on how to ensure the quality of BC services and how to develop programs and interventions to successfully improve outcomes of BC patients (Box 2).

In 2021, the WHO, in collaboration with the International Atomic Energy Agency (IAEA) and the IARC, [70] launched the GBCI which aims to provides national program managers, policymakers and multisectoral stakeholders the valuable guidance they need to assess gaps, strengthen and scale-up services for the early detection, diagnosis and management of BC [2]. The GBCI framework’s objective is to reduce BC mortality by 2.5% per year and save 2.5 million lives over a 20-year period [2]. For this purpose, the GBCI identified essential KPIs that countries should adopt to measure progress.

Box 2. **Overview of key initiatives driving quality improvement in BC care through evidence-based guidelines, scalable care models and clinical frameworks.**

The BHGI, founded in 2002, sought to improve breast health care and cancer treatment for women in economically disadvantaged countries, [66] by crafting and applying evidence-based guidelines, specifically developed and adapted for enhanced feasibility and contextual appropriateness [6, 7]. In collaboration with critical players, the BHGI defined best practices and created consensus guidelines for BC early detection, diagnosis, treatment, healthcare systems and public policy [6]. The BCI2.5, founded in 2014, aims to promote evidence-based care models for early detection, diagnosis, treatment and supportive care that can be scaled up when and where needed, providing regional leaders with analytics, assessment and planning tools, educational materials and implementation science research [8, 9]. Critical tools developed include Breast Health Care Assessment Questionnaire and Knowledge Summaries, which are used for baseline assessments and situation analyses, in partnership with countries or centers of excellence [8]. The BCI2.5 also developed a phased implementation model of an evidence-based framework to improve breast health care delivery in LMICs, which is being implemented in Brazil, Peru and Tanzania [67, 68].

Guidance from a strictly clinical perspective is provided through quality indicators outlined in various documents, including CPGs, integrated BC healthcare processes, clinical pathways and position papers [69]. This guidance is valuable not only for individual institutions but also serves as a framework for countries to assess and regulate the overall quality of BC services at the clinical level.

However, recent data show that only seven of the most developed countries are meeting, and six additional countries are close to meeting, the GBCI goal of 2.5% annual decreases in BC mortality rates [5]. This outlines a possible gap with countries requiring further support and guidance on a set of more practical and applicable essential measures to consider to more effectively and rapidly scale up action for improved BC care.

Building on the GBCI and previous global initiatives, the BCCQI proposes a unified framework that outlines essential elements for countries to prioritise in order to enhance BC care quality at the national level. By providing a structured set of targets and indicators, this framework enables countries to develop tailored implementation strategies, adapted to their specific contexts and create actionable roadmaps to address gaps in BC care.

Unlike rigid, prescriptive models or ‘one-size-fits-all’ approaches of the past, the BCCQI seeks to promote a flexible, context-sensitive approach, empowering countries to set their own short- and medium-term priorities. In doing so, countries are encouraged to leverage existing guidance from initiatives such as the GBCI and other resource-stratified frameworks. Recognising that no country has fully addressed all barriers to optimal BC care, the BCCQI helps nations identify specific areas for improvement. Some may focus on enhancing healthcare professionals knowledge and education, while others may work to improve access to supportive services that enhance patient-centered care. Because the framework is designed as a continuous, inclusive process, countries can refine their priorities and strategies iteratively, making stepwise enhancements based on local challenges and opportunities. In this way, countries with immediate limited investment capacities or differing priorities can still advance toward meaningful progress. This flexibility fosters a sense of ownership among national stakeholders, allowing each country to make strategic decisions aligned with their specific situation.

**Figure 2.**
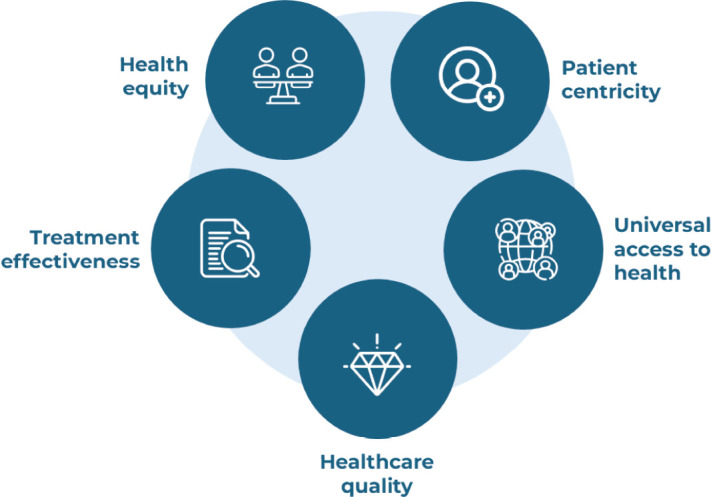
Domains for BC care improvement, as prioritised by the Breast Cancer Care Quality Index index (BCCQI).

In addition, to ensure that improvements are truly impactful for patients, the BCCQI centers on five key domains essential for high-quality BC care: health equity, patient centricity, universal health access, healthcare quality and treatment effectiveness (Figure 2 and Box 3).

By emphasising the importance of national policies and strategies, the BCCQI is designed to drive bold policy reforms that establish essential national frameworks. These frameworks will empower countries to sustainably strengthen healthcare system capacity while striving to keep pace with rapid advancements in BC clinical practice.

Given the scale of the challenge, no single stakeholder can drive this transformation alone. The BCCQI is intended to serve as a catalyst for collective action by uniting key actors around a comprehensive set of actionable targets and indicators. This shared framework helps expand and clarify responsibilities, enhances accountability and fosters a sense of ownership among national stakeholders when prioritising actions and measuring progress toward the GBCI goals.

Finally, over time, by supporting the harmonisation of progress tracking, as outlined by previous initiatives [71, 72], the BCCQI is expected to facilitate the generation of comparable global evidence. This will enable intercountry comparisons, trend identification and cross-country learning – critical information for policymakers seeking to refine existing frameworks and develop targeted interventions. Ultimately, this is expected to support decision-making, increased stakeholder engagement and advocacy and enhanced accountability, ensuring continued global progress toward the highest standards of BC care.

Box 3. **Definitions of the key domains for improvement prioritised by the Breast Cancer Care Quality Index (BCCQI).**

- Health equity: Describes the state in which everyone has a fair and just opportunity to attain their highest level of health. This involves removing obstacles to health such as poverty, discrimination and their consequences, including lack of access to good jobs with fair pay, quality education and housing, safe environments and health care [73, 74].
- Patient-centered care: Is achieved when services provided reflect the patient's preferences, needs and values [75]. This approach ensures that patient values guide program and service planning, as well as clinical decisions, emphasising the importance of effective communication, pain management, clear care plans and a comfortable environment.
- Universal health coverage (UHC): Means that all individuals and communities receive the health services they need without suffering financial hardship. It includes the full spectrum of essential, quality health services, from health promotion to prevention, treatment, rehabilitation and palliative care [76–79].
- Healthcare quality: Is the degree to which healthcare services for individuals and populations increase the likelihood of desired health outcomes. It encompasses six domains: safe, effective, patient-centered, timely, efficient and equitable care [80, 81].
- Treatment effectiveness: Is the likelihood that a certain treatment protocol will benefit patients in a certain clinical population when administered in clinical practice [82].

### Breast Cancer Care Quality Index

The BCCQI aims to increase action and implementation of healthcare system transformation to improve BC care for all patients, highlighting key elements that each country should focus on to advance the quality of BC care. The BCCQI represents a catalyst for engagement for policymakers, the international community and multisectoral stakeholders. Box 4 provides an overview of key principles that were followed during the conception of the BCCQI to ensure its robustness and maximise the likelihood of its successful adoption.

The BCCQI is structured into dimensions, goals, targets and indicators, mirroring the structure of key outcome-driven health-related frameworks established by international organisations and their collaborators. Box 5 provides more information regarding their development.

Each target and its supporting indicators have been designed to facilitate advances in the key domains of BC care improvement: health equity, patient centricity, universal access to health, healthcare quality and effectiveness of treatment (Figure 2 and Box 3).

Box 4. **Key guiding principles of the Breast Cancer Care Quality Index (BCCQI).**

(1) The BCCQI framework was developed in alignment with global health policy priorities and conventions to facilitate its adoption by policymakers (2). Through an inclusive collaboration and co-creation process, it ensures balanced representation and shared ownership among stakeholders, adhering to internationally recognised best practices (3). Designed to be both pragmatic and effective, the framework prioritises essential elements to enhance BC care quality globally. To promote fairness and equity, it emphasises inclusivity, ensuring that no population or individual is marginalised (4). Furthermore, to enhance its applicability across diverse settings, the framework has been built in consideration of a broad spectrum of evidence from various country contexts and income levels (5). Finally, the BCCQI is conceived as a flexible, iterative tool, allowing countries to adapt its implementation to their specific priorities and develop context-sensitive roadmaps.

Box 5. **Methodological information regarding the development of the goals, targets and indicators of the Breast Cancer Care Quality Index (BCCQI).**

The dimensions were identified through an in-depth review of the existing literature and are strongly supported by prior work on BC. The goals outline broad objectives, defining the desired achievements within each dimension. Targets specify measurable outcomes with defined timeframes, articulated through clear statements or quantitative benchmarks.

Indicators are the metrics used to monitor progress, assess gaps and measure advancement toward Targets. Achieving these targets is expected to help fulfil the overarching goals.

The dimensions are aligned to the three pillars of the GBCI framework: (1) early detection; (2) timely diagnosis and (3) comprehensive BC management. A fourth dimension that underlies the continuum of care and poses many challenges to the delivery of optimal BC services was strongly supported by the literature review findings. This dimension focuses on strong and resilient healthcare systems [2, 16].

The goals associated with each dimension were defined in accordance with the GBCI framework, the patient pathway presented in the GBCI framework and analysis of peer-reviewed literature.

The targets under each dimension were set and defined based on the analysis of each pillar during discussions with experts, the patient pathway presented in the GBCI framework and peer-reviewed literature [2, 16, 83, 84].

Finally, one or more indicators were built to translate each Target into measurable metrics. Indicators are categorised into ‘Structure, Process, Outcome’, following Donabedian's quality of care model [85], in which structure is defined by the attributes of the settings in which care is provided, process by the provision of care itself and outcome by the measurable change in the health status of the patient. Some adaptations of this model, shown in Table 2 below, were made to ensure the BCCQI is practical and easily implementable across countries, minimising hurdles that could impede its adoption.

As discussed above, when seeking to improve BC patients’ care, every country would need to identify its medium–short term priorities to advance specific targets and indicators of the BCCQI, based on the country context, including challenges and opportunities.

Table 3 summarises the BCCQI targets and indicators by dimension, including the domains each indicator contributes to enhancing. Additional information regarding each target and indicator is provided in the Supplementary Tables S1 and S2.

## Discussion

The BCCQI provides an opportunity to promote renewed attention and focus on health care and mobilising new streams of funds for BC care. It is envisioned as a dynamic tool, subject to continuous refinement and improvement based on real-world application and evolving evidence. As a flexible and adaptable framework outlining essential elements for assessing and improving the quality of care for BC patients across the care continuum, it promotes stakeholder ownership and accountability at all levels [86], empowering countries to drive iterative improvements through context-specific roadmaps aligned with local needs, opportunities and challenges. Moving beyond the prescriptive, rigid models of the past, it encourages action through an inclusive and continuous process that recognises incremental progress. This allows countries to address public health challenges at their own pace and has the potential to serve as a replicable model for advancing care in other critical disease areas. Ultimately, this approach brings countries closer to fulfilling the three pillars of the GBCI framework, providing additional guidance and more practical and applicable details to help them reduce BC mortality and address its broader societal impact.

The essential domains that guided development of the BCCQI serve to enhance alignment of the framework with the international community’s global health priorities, like as example, the 2030 Agenda for Sustainable Development. Additionally, in the lead up to the fourth UN high-level meeting on noncommunicable diseases (NCDs), the BCCQI presents an opportunity for the cancer and BC communities to push for reprioritisation of an unfinished agenda. The marked alignment of the BCCQI with other global health priorities, such as universal health coverage and non-communicable disease management, guarantees that policymakers can more easily secure the necessary political and financial support due to this synergy with ongoing efforts at the country and global level.

As seen in previous disease-specific programs, foundational frameworks can often serve as a platform for broader service integration, yielding benefits that extend beyond their original scope [86–88]. Similarly, the BCCQI framework has the potential to generate positive spillover effects that enhance the overall strength and resilience of national healthcare systems. This is particularly relevant for the BCCQI, which directly focuses on overall healthcare system strengthening by dedicating a full dimension to integral healthcare system components.

By incorporating metrics related to governance, financing, resource allocation, service organisation and results, this framework is fully geared towards ensuring that impact and advances in overall healthcare system capacity can be systematically recorded and monitored. Monitoring and evaluation should indeed become a pivotal component of any BC planning and implementation effort, as outlined by previous initiatives [71, 72]. The fact that the BCCQI already provides a general reference aids countries in setting a starting point to begin recording the baseline, in support of BC and healthcare system performance assessment and improvement over time.

Yet, several challenges must be understood and mitigated to ensure the successful adoption of the BCCQI. First and foremost, the perception that BC is an already addressed need might reduce the willingness of policymakers to continue working on it.

In addition, the inadequate public awareness and the lack of robust advocacy movements further hinder the prioritisation of BC as a key health issue. There is also a lack of comprehensive data, as many countries face gaps in national cancer staging, timely diagnosis and treatment completion data, which leads to a lack of agreement on critical aspects of BC care. More specifically, for example, in LMICs, there are critical evidence gaps in building the case for comprehensive BC management services, especially when considering the full spectrum of the desirable supportive services to be offered. These gaps pose remarkable challenges for countries with limited capacity to invest in data infrastructure development in the short-medium term but, on the other hand, offer them the opportunity to leverage the index as a tool for broader awareness creation, resource mobilisation and collaborative action for iterative progress.

Finally, financial, infrastructural and human resource constraints, along with competing priorities within and beyond healthcare systems, pose substantial barriers to the adoption of the BCCQI.

As we look ahead, sustained collaboration among governments, the international community and multisectoral stakeholders will be essential to driving continuous progress in addressing growing healthcare needs worldwide. By offering a unified framework for action and fostering inclusivity and adaptability to support decision making, the index empowers countries and multisectoral stakeholders to develop and advance tailored efforts to bridge gaps in BC care. Collaboration will be especially valuable in advancing data infrastructure needs and pilot programs to field test and further refine the BCCQI, which is conceived as a dynamic tool that, once adopted, will be subject to ongoing refinement and future improvements based on real-world application and evolving evidence. In this regard, key opportunities for immediate action, which are ultimately expected to have positive repercussions on the broader healthcare system and global health, have been pinpointed in the recommendations.

## Limitations

The methodology used in this study, which includes a review of peer-reviewed literature, grey literature, UN resolutions and frameworks and an expert feedback process, guided the development of the BCCQI as an expert-endorsed tool designed to drive actionable, systematic healthcare transformation and improve the quality of BC care for all patients. However, the HIC-bias of grey literature and CPGs may represent a limitation of the tool.

This geographic concentration may not fully capture the unique challenges and resource constraints faced by LMICs. However, the inclusion of peer-reviewed literature from countries across diverse regions and development levels helps mitigate this limitation by incorporating a broader range of perspectives. Future iterations of the BCCQI could further enhance its comprehensiveness and generalisability through a systematic review of the existing evidence.

The expert feedback process, although robust and multidisciplinary, reflects the perspectives of a selected group of professionals. While this could potentially limit the generalisability of findings, the experts consulted for this study were carefully chosen based on their affiliations and roles within reputable organisations dedicated to BC, extensive experience in oncology and related fields and active engagement and participation in BC policymaking and guideline development.

Moreover, their representation from a diverse range of geographic locations and healthcare systems reinforces the global applicability of the BCCQI.

Finally, while the metrics outlined in the BCCQI are derived from evidence, they have not been field-tested. The primary goal of this study is to initiate meaningful discussions on the development of the BCCQI as a global tool to be adopted in NCCPs. It is intended to represent, despite its limitations, a first attempt at developing such a framework at the global level and to lay the foundation for following iterations as more evidence becomes available. However, it is important to recognise that incorporating the BCCQI into NCCPs does not guarantee better patient outcomes and higher quality care delivery, as clear evidence of the impact of similar frameworks in key governance documents at the national level is still missing.

## Recommendations

The BCCQI serves as a catalyst for unifying stakeholder efforts and accelerating global progress in BC care by building on existing initiatives and maximising the impact of prior investments. It offers a clear, actionable and adaptable framework focused on key dimensions, including early detection, timely diagnosis, comprehensive management and healthcare system resilience. However, while adopting as part of NCCPs and implementing the BCCQI stands as a key recommendation, existing challenges may hinder global commitment to its success. To this end, policymakers, international organisations and stakeholders across sectors play a critical role in building momentum and fostering broad engagement toward a strong global partnership striving to guarantee that the BCCQI becomes a pivotal tool to ensure that all BC patients have access to the highest standards of care.

To advance this objective, the following recommendations are proposed (Box 6).

Box 6. **Recommendations.**

**Recommendations for policymakers**

- **Leverage upcoming international platforms to advocate for renewed international commitment to the unfinished BC agenda:** Maximise opportunities created by major global gatherings, such as the UN high-level meeting on NCDs, to elevate the unfinished BC agenda and garner support for implementation of the BCCQI.
- **Support integration of the BCCQI into National Cancer Control Plans by emphasising the BCCQI’s alignment with the UN global health agenda and specific sustainable development goals, such as SDG 3.4** *(reducing premature mortality from NCDs)* **and SDG 3.8** *(achieving universal health coverage):* Emphasise the BCCQI’s alignment with the UN global health agenda and specific sustainable development goals, such as SDG 3.4 *(reducing premature mortality from NCDs)* and SDG 3.8 *(achieving universal health coverage)*, and encourage governments to champion a UN-led initiative to improve BC care globally by integrating the BCCQI into NCCPs.
- **Promote cross-sector and supranational collaboration for BCCQI implementation to tackle key barriers, including resource allocation, data collection and monitoring systems:** Foster alliances among governments, NGOs, healthcare institutions and the private sector to leverage expertise and mobilise the resources needed for the implementation of the BCCQI.
- **Prioritise BC public education and awareness to create the demand for adoption and implementation of the BCCQI:** Promote comprehensive awareness programs and campaigns to reduce stigma and fear surrounding BC. Leverage existing platforms and channels, like advocacy networks and international days and events, to amplify public support for improved BC care and increase endorsement for the BCCQI.
- **Build the economic case for investment in coordinated global efforts to improve BC care, reduce inequities and promote new funding stream:** Work with multisectoral stakeholders to generate the necessary evidence in support of continued investments in BC care and mobilise both traditional and innovative funding streams to facilitate the implementation of the BCCQI.
- **Support phased national adoption of the BCCQI by developing context and resources -specific pathways for implementing the BCCQI:** Develop context-specific pathways for implementing the BCCQI. Prioritise key areas of improvement based on available resources, opportunities and challenges, and gradually expand efforts as capacity grows. When necessary, set up pilot programs to test feasibility and scalability before nationwide rollout.

**Recommendations for the international community**

- **Leverage the BCCQI to galvanise Member States and trigger renewed actions for improved BC care quality in support of the GBCI framework:** Build on stakeholders’ activities and harness the momentum of the BCCQI to ignite enthusiasm and drive faster progress in improving BC care quality worldwide. Foster collaborative actions and interactions, encouraging cross-pollination between WHO/UN-led initiatives and externally driven efforts as a model for action that could be expanded to other disease areas in the future.
- **Strengthen global coordination, knowledge sharing and synergies with the WHO initiatives:** Foster collaboration among stakeholders by aligning efforts with the GBCI framework and leveraging key resources such as the WHO Patient Navigation Toolkit, the WHO Noncommunicable Disease Facility-Based Monitoring Guidance and the WHO–IAEA Framework for Setting Up a Cancer Centre. Actively encourage stakeholders and Member States to maximise these tools while promoting international knowledge-sharing platforms to facilitate peer-to-peer learning, avoid duplication of efforts and accelerate country progress in BCC quality worldwide.
- **Promote renewed focus and attention on BC across upcoming global platforms:** Ensure that upcoming global appointments, such as the UN High-Level Meeting on NCDs, prioritise discussions on the unfinished BC agenda. Advocate for dedicated attention to BC care, emphasising to Member State its urgency and alignment with key commitments, including the 2030 Sustainable Development Agenda—particularly SDG 5 (*gender equality*) and SDG 10 (*reducing inequalities*).
- **Support member States’ initiatives and actions to accelerate country-level progress on improved quality of BC care delivered:** Offer support and provide technical assistance for countries seeking to adopt and implement the BCCQI. Promote the development of research networks and collaborations that focus on scaling successful models and monitoring BCCQI implementation.
- **Enhance international and multisectoral cooperation for resources mobilisation and expansion:** Promote cross-sector partnerships to address key barriers related to resource availability, by fostering collaborations between governments, NGOs, healthcare institutions and the private sector. Promote the expansion of traditional funding initiatives and facilitate the exploration of innovative solutions to support countries in implementing the BCCQI, with particular focus on low- and middle-income countries.
- **Support research and country-level evidence generation to accelerate progress in the quality of the BC services delivered:** Strengthen efforts to improve data collection systems, emphasising the importance of investments in robust and sustainable data gathering and analysis mechanisms to measure progress against BCCQI indicators and identify gaps and disparities to generate actionable, context-specific evidence and inform policy and program evaluation and adaptation.
- **Assist BC stakeholders’ monitoring and evaluation efforts:** Build on existing international data gathering and monitoring initiatives, like IARC CanScreen 5 and IARC CanReg 5, to expand BC related data gathering and analysis. In support of these initiative, consider the establishment of an international multistakeholder mechanism, modeled after the Global Coordination Mechanism on NCDs, to enhance commitment and global accountability for standardised data reporting to enable inter-country comparisons and generate global evidence on BC care.

**Recommendations for multisectoral stakeholders**

- **Scale up advocacy towards policymakers for enhanced efforts to improve BC care and adoption of the BCCQI:** Advocate at the national level to ensure that BC remains or becomes a priority. Highlight the significant gaps, the growing burden and the societal impact and cost of BC, supporting policymakers in making the case for continued investment, even in well-resourced settings. Urge adoption and implementation of the BCCQI as a way to improve the quality of BC care provided at the national level and globally.
- **Join forces at the international level to build momentum for the BCCQI and renewed BC attention:** Collaborate with partners at regional and global levels to ensure visibility of the current gaps and challenges faced by BC patients and in providing quality BC diagnosis and management. Use discussions and side events of key fora, like for example the World Health Assembly, the UN General Assembly and the UN High-level Meeting on NCDs, to raise awareness about the need to ramp up efforts for BC and foster international collaboration for a joint UN-resolution on BC and the adoption of the BCCQI.
- **Foster support and commitment for BC and the BCCQI as part of broader global health and sustainable development efforts:** Emphasise how the BCCQI aligns with the broader sustainable development agenda – especially with SDG 3.4 *(reducing premature mortality from NCDs)*, SDG 3.8 *(achieving universal health coverage),* SDG 5 *(gender equality)* and SDG 10 *(reducing inequalities)* – as well as with prominent ongoing efforts by the WHO and other UN-related agencies, e.g., the GBCI framework, IARC CanScreen 5 and CanReg 5. Call upon the international community to continue advancing initiatives that promote inter- and intra-country equity in BC care.
- **Participate in international platforms and collaborative initiatives:** Support efforts championed by the international community to promote the BCCQI by contributing with expertise, information and resources as needed. For example, commit to support standardised data reporting from the country level to key global BC-related initiatives and platforms, like for example IARC CanScreen 5 and CanReg 5.
- **Expand advocacy, education and capacity-building efforts in support of BC awareness and BCCQI implementation:** Intensify advocacy and capacity-building initiatives directed toward the general public, peer advocates and healthcare workers at the regional and national levels. Strengthen awareness, build support and activate grassroots movements for the adoption and implementation of the BCCQI as a way to reduce disparities and improve BC outcomes locally and globally.
- **Leverage synergy and alignment with other initiatives to expand efforts and partnerships:** Collaborate with partners and stakeholders at every level to integrate BC care into broader health system strengthening and development efforts, such as those focused on universal health coverage, non-communicable disease management and addressing global inequities.
- **Strengthen evidence-generation efforts to monitor and evaluate progress in the quality of BC care:** Leverage collective expertise, technological resources and financial capacity to build a compelling case for scaling up initiatives. Learn from successful examples of previous and ongoing efforts, such as the global burden of disease (GBD), Globocan and Concord, to promote coordinated and synergistic evidence-generation activities at both regional and global levels.
- **Engage financial actors to support effective fund mobilisation:** Foster discussions with economic institutions and financial stakeholders to explore new opportunities for resource mobilisation, including innovative financing mechanisms and successful initiatives. Support policymakers and the international community in their efforts to mobilise and expand resources for BC care and implementation of the BCCQI.

## Conclusion

Significant disparities in BC access and quality of care persist globally, including within HICs, highlighting the urgent need for a unified framework that supports all countries in advancing BC care, regardless of their context, resources, infrastructure or specific challenges. The Breast Cancer Care Quality Index (BCCQI) offers countries the guidance they need to design results-driven approaches supported by evidence to tackle their respective challenges at their own pace.

The global action to combat BC calls for coordinated and effective measures that streamline efforts, reduce unnecessary duplication and address existing gaps in care. In this spirit, it is important to emphasise that the BCCQI has been thoughtfully designed to further support and strengthen action under the GBCI and other relevant initiatives by providing more detailed metrics that countries can adopt in a context-specific manner. Rather than competing, it acts as a valuable tool to help operationalise the general guidance applicable to broad country groups provided by existing initiatives such as the GBCI framework, deepening their impact by empowering countries to develop their own tailored roadmaps for implementation.

In the lead-up to the UN high-level meeting on NCDs in September 2025, this framework presents an opportunity to drive renewed attention to BC care and mobilise new funding streams to enhance its quality at the global level. However, the successful adoption and implementation of the BCCQI requires multisectoral collaboration and coordinated action to overcome multiple barriers. It is envisioned that, by establishing clear benchmarks to be tracked through regular audits for the refinement of its implementation, integration of the BCCQI into NCCPs can enable more systematic efforts towards strong governance, sustainable financing and robust data collection and analysis systems.

Future studies can explore the implementation process of the BCCQI, and field-test the index to refine it, validate its metrics and ensure its relevance and reliability across different contexts. This will help make the BCCQI more evidence-based and context-validated.

By providing a tool for coordinated action at the global, national and regional levels, the BCCQI has the potential to serve as a catalyst for enhanced efforts towards more equitable and sustainable BC care worldwide. Its adoption and effective implementation are expected to lead to higher quality, patient-centered services globally, ultimately improving BC care for all patients, regardless of geographic or economic barriers.

## Conflicts of interest

BOA declares consulting fees from AstraZeneca as Global Oncology Consultant (2024–25).

GC declares consulting fees from BMS, Roche, Pfizer, Novartis, Lilly, AstraZeneca, Daiichi Sankyo, Merck, Seagen, Ellipsis, Gilead and Menarini; is a speakers’ bureau member for Lilly, Pfizer, Relay, Gilead, Novartis and Menarini; and received support for attending meetings/travel from Daiichi Sankyo.

SS received speaking fees and participated in advisory boards for Novartis, Gilead, Lilly, Roche, AstraZeneca, Daiichi Sankyo, Knight, Merck, Bristol Myers Squibb, Incyte and Sandoz.

FC holds consultancy roles with Amgen, Astellas/Medivation, AstraZeneca, Bayer, Celgene, Daiichi Sankyo, Eisai, GE Oncology, Genentech, Gilead, GlaxoSmithKline, IQVIA, MacroGenics, Medscape, Merck Sharp & Dohme, Merus BV, Mylan, Mundipharma, Novartis, Pfizer, Pierre Fabre, prIME Oncology, Roche, Sanofi, Samsung Bioepis, Seagen, Teva and TouchIME; is the President of the ABC Global Alliance, Chair of the ABC Consensus Conference and Guidelines, and a Fellow of the European Academy of Cancer Sciences; and is a member or committee member of ESMO, ESO, ASCO, AACR, ECO, AORTIC, UICC, EORTC, IBCSG, SOLTI, EACR, SIS, ASPIC, SPO and EVITA.

ARG reports being the CEO of Policy Wisdom LLC.

ES received travel grants from Boehringer Ingelheim, Hikma, Janssen, Lilly, Merck Sharp & Dohme, Mundipharma, Novartis, Pfizer and Roche; participated in advisory board meetings, consultancy and/or speakers’ bureaus for Amgen, AstraZeneca, Bayer, Boehringer Ingelheim, Clinart MENA, Genomics Genetics, GSK, Hikma, EVA Pharma, Janssen, Lilly, Merck Sharp & Dohme, Mundipharma, Nebridge Pharmaceutical, Novartis, Pfizer, Pierre Fabre, Roche and Sandoz; and has been involved in clinical trials for AstraZeneca, Boehringer Ingelheim, Novartis, Pfizer and Roche.

CHY declares payment or honoraria for lectures, presentations, speakers’ bureaus, manuscript writing or educational events from Novartis; and participation on a Data Safety Monitoring Board or advisory board for AstraZeneca.

AM declares consulting fees from Roche and Eli Lilly; payment or honoraria for lectures, presentations, speakers’ bureaus, manuscript writing or educational events from Merck, Roche, Eli Lilly and AstraZeneca; and received support for attending meetings and/or travel from Roche.

YCG received research support from Roche and Pfizer; participated in speakers’ bureaus for Gilead, AstraZeneca, Lilly and Merck Sharp & Dohme; received support for travel expenses from Novartis, Pfizer, Gilead and Lilly; and holds an advisory role with AstraZeneca.

EC, MM, YY, JVR, IL and NS declare no competing interests.

EC, BOA, GC, SS, FC, ES, AM, YCG and YY received honoraria for their involvement as expert panel members. MM’s honorarium was allocated in support of the Kenya Society of Hematology and Oncology (KESHO). The participation of JVR, IL and NS was covered through their regular roles at Policy Wisdom LLC.

## Author contributions

- Conceptualisation- Formulation: IL, NS.
- Conceptualisation- Evolution: EC, BOA, GC, SS, FC, ARG, ES, CHY, AM, YCG, MM, YY.
- Data curation: JVR, IL.
- Formal analysis, data adaption to real world experience, data interpretation: EC, BOA, GC, SS, FC, ARG, ES, CHY, AM, YCG, MM, YY, JVR, IL, NS.
- Investigation: EC, BOA, GC, SS, FC, ARG, ES, CHY, AM, YCG, MM, YY, JVR, IL, NS.
- Methodology- Original: JVR, IL, NS.
- Methodology- Refinement and adaptation: EC, BOA, GC, SS, FC, ARG, ES, CHY, AM, YCG, MM, YY.
- Index Endorsement: EC, BOA, GC, SS, FC, ARG, ES, CHY, AM, YCG, MM, YY.
- Project administration: NS.
- Supervision: NS.
- Writing – original draft: JVR, IL.
- Writing – review & editing: EC, BOA, GC, SS, FC, ARG, ES, CHY, AM, YCG, MM, YY, NS.

## Data sharing statement

Not applicable.

## Figures and Tables

**Table:** 

Term	Definition
Accessible	To be considered an accessible healthcare service, three criteria must be met—physical accessibility, financial affordability and acceptability [1–3]. Acceptability represents a person’s willingness to seek services. This can be positively or negatively influenced by social and cultural factors such as language, age, sex, ethnicity or religion of the health provider [2].
Adequate/ Comprehensive multidisciplinary treatment/Care	Treatment/care is considered multidisciplinary when it is planned by a team of multidisciplinary healthcare professionals. According to the World Health Organization (WHO), a cancer multidisciplinary board (MTB) must include radiologists, pathologists, surgical oncologists, medical oncologists, radiation oncologists and healthcare professionals specialized in supportive oncology [4]. Multidisciplinary Treatment/Care is considered adequate when it is planned in accordance with international guidelines’ recommendations, and comprehensive when it involves a team of team of multidisciplinary healthcare professionals identified by WHO.
Adherence	The extent to which a person’s behaviour corresponds with agreed recommendations from a healthcare professional [5].
Adoption	The process by which an entity, such as a government, organization or institution, decides to implement, approve or put into practice a specific policy, program, law, regulation or measure.
Affordable	Care that is accessible to patients as needed, without causing financial hardship. Costs may be associated, but they must not create barriers that delay or deter access [6, 7].
Appropriate	Interventions aligned with international guidelines and aimed at improving breast cancer patient outcomes and quality of life and maximizing survival rates.
Appropriate diagnosis	A diagnosis is considered appropriate if it is accurate, initiated promptly and completed within 2 months of the patient's first presentation with a suspicious finding. It must follow a comprehensive, multi-step process to fully characterize the disease [8] and determine its presence, type and extent—aligning with the definition of a *complete diagnosis* (see below).
Complete diagnosis	Comprehensive multi-step diagnostic process, including clinical evaluation, imaging, tissue sampling, pathological analysis, HR/HER2 testing and germline genetic testing when indicated and available.
Comprehensive information	Comprehensive information on breast cancer includes all data necessary to guide patient-centered, multidisciplinary treatment, support clinical decision-making and advance research on etiology, epidemiology, treatment response and population variations. This encompasses: i. Age at diagnosis, ii. Gender, iii. Ethnicity/race (whenever possible), iv. Geographic location, v. Stage at diagnosis, vi. Tumour subtype, vii. Family history, viii. Known molecular markers and mutation, ix. Type of all treatments, x. Date of all treatments, xi. Recurrence, xii. Information on recurrence (local, regional and/or distant) and xiii. Outcome.
Dissemination	Purposeful distribution of information and materials to a specific clinical practice audience [9].
Early diagnosis program	Intervention intended to detect conditions as early as possible among people with symptoms, in contrast to screening programs that focus on asymptomatic individuals [10].
Elevated risk population	A high-risk population for breast cancer, identified based on country-specific risk assessment approaches. Some countries may define high risk based on age, family history and personal history, while others may adopt more comprehensive assessment methods.
Equitable	Services delivered in a way that ensures fairness, minimizing avoidable or remediable disparities in health status across population groups, regardless of social, economic, demographic or geographic factors [11].
Framework	A structured approach that provides a comprehensive representation of key factors, concepts and variables to explain and guide policy implementation and understanding [12].
Healthcare Workers/Workforce	A diverse group of people who work to improve health, including doctors, nurses, midwives, public health professionals, laboratory technicians, health technicians, medical and non-medical technicians, personal care workers, community health workers, healers and traditional medicine practitioners [13].
Health literacy	Personal knowledge and competencies that accumulate through daily activities, social interactions and across generations. Personal knowledge and competencies are mediated by the organizational structures and availability of resources that enable people to access, understand, appraise and use information and services in ways that promote and maintain good health and well-being for themselves and those around them [11].
International guidelines	A document developed, adopted and regularly updated by internationally recognized organizations (e.g.,* ASCO, ESMO* and* NCCN)*^1^ that contains recommendations for clinical practice or public health policy. It tells the intended end-user of the guideline what they can or should do in specific situations to achieve the best health outcomes possible, individually or collectively, by offering a choice of different interventions or measures that have an anticipated positive impact on health and implications for the use of resources [14].
National policy	A comprehensive framework that outlines a country’s vision, policy direction and strategies for achieving specific objectives. National health policies aim to ensure population health [15].
Optimal coordination	Care coordination synchronizes the delivery of a patient’s health care from multiple providers and specialists. The goals of coordinated care are to improve health outcomes by ensuring that care from disparate providers is not delivered in silos, and to help reduce healthcare costs by eliminating redundant tests and procedures. It includes easy access to a range of healthcare services and providers; good communication and effective care plan transitions between providers; focus on the total healthcare needs of the patient; and clear and simple information that patients can understand [16].
Patient engagement	The process of facilitating and supporting the active involvement of patients in their own care, in order to enhance safety, quality and patient-centeredness of healthcare service delivery through platforms for participation in healthcare decision-making and health service planning and design [17]. Patient engagement can be promoted through different types of *platforms for participation* (see below).
Patient navigation	A strategy designed to overcome individual- and system-level barriers to timely diagnosis and treatment. It aims to facilitate timely access to healthcare services that enable and empower breast cancer patients to access required information and services at cancer centers, while promoting healing and incorporating support for both patients and caregivers [18, 19].
Platforms for participation	Platforms for participation are mechanisms to enable patients, families and caregivers to contribute to the design or development of patient-centered processes and systems. Examples include advisory committees, systematic open consultations with patient groups, surveys, online feedback channels, interviews and focus group discussions [17].
Prompt/Timely access	Timely availability of appropriate healthcare services without unnecessary delays, ensuring patients receive the right care at the right time. Prompt/timely access means that individuals can receive preventive, diagnostic, treatment and follow-up services as soon as needed, minimizing wait times that could compromise health outcomes.
Quality services	Quality of care is the degree to which health services for individuals and populations increase the likelihood of desired health outcomes and are consistent with current professional knowledge. To support delivery of high-quality health care, services should be effective, efficient, safe, integrated, equitable, timely and patient-centered [20].
Resource-appropriate guidance	Guidance developed at the country level in alignment with international evidence-based, resource-stratified clinical practice guidelines and in consideration of the specific country context and available resources.
Service integration/Patient navigation mechanisms (well-defined)	Structured systems that a country implements to ensure seamless integration of healthcare services, guiding patients through the healthcare continuum from screening and diagnosis to treatment, follow-up and survivorship care. Key components of these mechanisms can include care coordination networks, patient navigators, centralized referral and tracking systems, health information systems integration and community-based support and education [18]. To be considered well-defined, it mechanisms should be established through national or subnational regulatory and policy frameworks that aim to eliminate barriers to care, improve coordination and enhance patient outcomes.
Specialized diagnostic services	Advanced methods used to detect, diagnose and stage breast cancer. These services go beyond standard clinical exams and may include imaging techniques (e.g., mammography, ultrasound and MRI), biopsy procedures (e.g., fine needle aspiration or core biopsy) and molecular testing (e.g., genetic markers, hormone receptor status) to assess the type, stage and genetic profile of a tumour. Specialized diagnostic services are critical for tailoring personalized treatment plans and improving patient outcomes.
Specialized settings	Healthcare facilities with specialized staff and equipment to provide advanced diagnostic services and multidisciplinary treatment and care.
Supportive medications	Medications that are an essential part of supportive care and used to prevent and manage the physical and psychosocial adverse effects of cancer and its treatments. Along with preventive medications, they help reduce the risk of serious complications, such as infections. As part of supportive services, these medications play a key role in comprehensive breast cancer care, as failure to address adverse effects and complications can lead to decreased treatment adherence, intervention failure and poorer patient outcomes [4, 21].
Supportive services	Services that help cancer patients, their caregivers and their families to cope with the disease and its treatment throughout the patient's pathway and to help the patient maximize treatment benefits in order to cope in the best possible way with the effects of the disease [22].
Survivorship care	Ongoing care provided to patients after cancer treatment, focusing on surveillance for recurrence, management of long-term and late effects, psychosocial support and patient education on self-management and wellness. It transitions from active treatment to health maintenance, promoting healthy behaviours and monitoring for potential recurrence.
Suspicious findings	Findings from a clinical breast examination are suggestive of breast cancer and require specialized diagnostic services for confirmation and a complete diagnosis.
Treatment completion	The completion of all components or steps of the prescribed therapeutic regimen, except in cases where interruption is medically indicated and recommended by a qualified healthcare professional responsible for the patient's care [4].
Treatment abandonment	Failure to complete all components or steps of the prescribed therapeutic regimen for reasons other than a medically indicated interruption that is recommended by the healthcare professional responsible for the patient's care [4].

1 American Society of Clinical Oncology (ASCO); European Society for Medical Oncology (ESMO); National Comprehensive

**Table 1.** Overview of key challenges identified in BC detection, diagnosis and care.

**Early BC detection**
Behavioural and psychosocial factors (stigma, fear, concerns about fertility, gender inequity, low health literacy and common misconceptions that cancers are contagious or always fatal) as significant drivers of help-seeking interval (time between when a woman first notices symptoms of cancer and when they seek medical care) [2, 28, 33–38]. Accessibility of primary care facilities, including inability to obtain appointments and lack of transportation [28]. Suboptimal knowledge of BC and skills among healthcare professionals, disproportionately affecting specific groups (e.g., black, Asian and younger women) [2, 25, 26, 33, 39–43]. Limited use of quantitative tools that assess BC risk factors beyond family history, due to insufficient education/training and perceived discomfort [39, 44]. Limited access to mammography, in LMICs, due to high costs, low affordability, equipment shortages and lack of skilled practitioners [34, 43].
**Timely BC diagnosis**
Lower socioeconomic status, lower income, [45] and being unemployed [28, 46]. Distance from healthcare facilities [45]. Low health literacy and disease awareness [28, 46, 47]. Stigma and negative perception of treatment options, prognosis [46, 48]. Pursuance of complementary or alternative therapies before obtaining formal medical diagnosis [46]. Being unmarried, possibly due to limited support networks or financial resources [28, 46]. Lack of support or obstructive behaviour from family members and resistance to opposite‐gender examinations (i.e., male physicians performing breast examinations) [28, 46]. Type of healthcare facility, e.g., individuals seeking care in public/government-subsidised facilities have been reported to experience longer diagnostic intervals [36, 46]. Disease-related factors, specifically intrinsic to the condition and its manifestations, e.g., site, size, signs, symptoms and growth [46].
**Comprehensive BC management**
Low educational levels [49] and lack of formal employment, which disproportionately affect vulnerable populations in LMICs [28]. Geographic barriers, [32, 50] including rural residency [28]. Low health literacy and disease awareness [49]. Disease misconception, including fear of the disease or its treatment, and concerns about recurrence and impacts on daily activities [51]. Insufficient coverage, [49] unaffordable out-of-pocket expenses [2]. Resource constraints, referral delays and long waiting times [32]. Misdiagnosis by healthcare professionals [32]. Low access, poor provision and limited awareness of supportive care services and misconceptions about their use [50, 51]. Poor communication with healthcare professionals, perception of a negative attitude or bad interpersonal experience [32]. In LMICs, geographical and financial barriers, resource shortages and the unavailability of specialists [32, 50]. In LMICs, lack of consideration for patient-reported outcome measures (PROMs) [52].
**Overarching healthcare system**
Weak governance limits access to quality BC care, as many countries lack national cancer plans, essential medicines and diagnostic resources [61]. Limited national insurance coverage, particularly for economically disadvantaged populations [53]. Absence of sustainable financing mechanisms to set up dedicated national programs [2, 54, 55]. Lack of dedicated funding for essential diagnostic and treatment services, particularly in LMICs [32, 34]. Weak referral systems and poorly coordinated care [5, 28, 32, 46]. Limited geographical accessibility [5, 28, 32]. Unavailability of advanced pathology services, essential for accurate BC diagnosis and staging [56, 57], as well as a shortage of radiation therapy units, especially in Africa [58]. Shortage of healthcare professionals and trained specialists (e.g., pathologists, oncologists, radiologists and specialised nurses), particularly severe in LMICs [32, 46]. Deficiencies in medical products and technologies, with many healthcare facilities lacking essential diagnostic and treatment equipment, such as mammography machines [32, 34]. Inconsistencies in adherence to clinical practice guidelines (CPGs). [59–61]. Long waiting times and appointment cancellations [32]. Weak health information systems, including inadequate cancer registries, incomplete data collection and insufficient monitoring mechanisms, which are more prominent for specific groups (e.g. metastatic BC patients) [16, 32].

**Table 2.** Description of the categories of indicators adopted.

Structure	Qualitative indicators that seek to capture existing policies, programs, strategies, legislation and guidelines.
Process	Quantitative indicators adapted from indicators found in peer-reviewed literature.
Outcomes	Quantitative indicators aligned with the KPIs presented by the GBCI framework and supported by the literature review. These focus on outputs, serving as practical measures of the progress towards the desired outcome, i.e., improving the quality of BC care.

**Table 3.** Overview of the Breast Cancer Care Quality Index (BCCQI) targets and indicators by dimension, including information regarding the domains supported by each element. (Continued)

Dimension identification, dimension, and goals	Target/Indicator Identification	Domains	Target/Indicator
A. Early breast cancer detection *Promote early detection of breast cancer*	**A.1**		Establish appropriate national programs, policies or frameworks to ensure availability of and equitable access to affordable and appropriate BC early detection programs and services.
	A.1.1	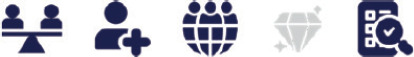	Country has a national program, policy or framework that sets out appropriate measures to ensure availability of and equitable access to affordable and appropriate primary health care or community BC early detection programs and services, where individuals with suspicious findings are identified and referred for diagnostic work-up.
	A.1.2	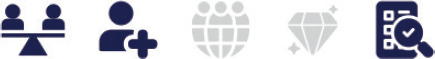	Breast cancer awareness and education programs for the public and healthcare workers to support early detection and increase recognition of early signs and/or symptoms of BC are included in national health-related frameworks.
	**A.2**		Ensure availability of and access to affordable and appropriate BC early detection programs and services.
	A.2.1	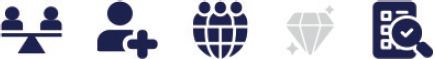	Country establishes and guarantees the execution of affordable and appropriate primary health care and community BC early detection programs and services for the identification and referral of individuals with suspicious findings for diagnostic work-up.
	A.2.2	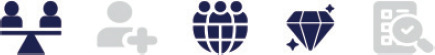	Proportion of women at elevated risk of BC screened at least once every 2 years.
	**A.3**		Ensure that at least 60% of invasive BCs are diagnosed at stage I or II.
	A.3.1	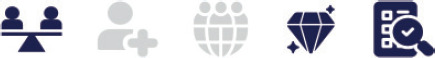	Proportion of invasive cancers diagnosed at stage I or II according to TNM anatomic and/or pathological staging.
B. Timely breast cancer diagnosis *Ensure timely access to appropriate breast cancer diagnosis*	**B.1**		Ensure timely and equitable access to affordable and quality diagnostic services.
	B.1.1	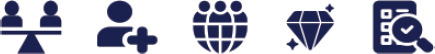	Country has a national policy or framework that guarantees prompt access to equitable, accessible, affordable and quality diagnostic services in specialised settings after a suspicious finding.
	**B.2**		Ensure diagnosis completion (including clinical assessment, imaging, tissue sampling, pathological analysis, HR/HER2 testing) within 2 months from first access/presentation due to a suspicious finding.
	B.2.1	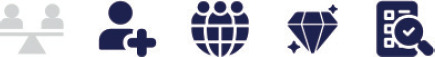	Proportion of patients with complete and appropriate diagnosis and staging (including clinical evaluation, imaging, tissue sampling, pathological analysis, HR/HER2 testing and germline genetic testing when indicated and available) within 2 months from first access/presentation due to a suspicious finding.
C. Comprehensive breast cancer management Guarantee timely access to comprehensive breast cancer treatment and care for all patients at all stages	C.1		Ensure timely, equitable and affordable access to quality, comprehensive and multidisciplinary BC care and management, including supportive services like pain management, physiotherapy, supportive medications, lymphedema management, psycho-oncology and oncofertility.
	C.1.1	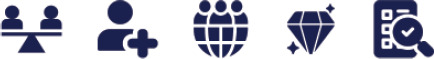	Country has a national policy or framework to guarantee timely, equitable and affordable access to comprehensive multidisciplinary care, from treatment initiation to completion.
	C.1.2	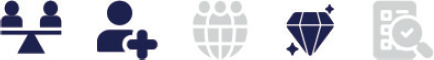	Proportion of patients with confirmed diagnosis of breast cancer who have their adequate multidisciplinary treatment initiated within 3 months from first access/presentation due to a suspicious finding.
	C.1.3	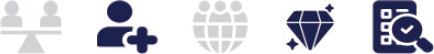	Proportion of triple-negative BC, HER2+ and HR+/HER- early BC patients that receive neoadjuvant therapy or primary surgery as their first treatment, in alignment with what is recommended by international guidelines.
	C.1.4	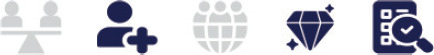	Proportion of patients with hormone receptor-positive invasive BC who received endocrine therapy (ET) in alignment with international guidelines.
	C.1.5	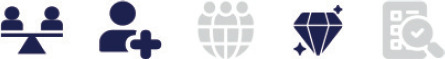	Proportion of patients that receive adequate supportive services and palliative care they need, e.g., pain management, physiotherapy, supportive medications, lymphedema management, psycho-oncology support and oncofertility, out of the total number of patients in treatment.
	C.1.6	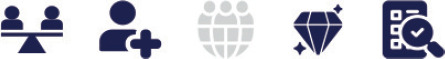	Country has provided guidance or is working towards the incorporation of patient perspective in its service quality assessment protocols for BC within the infrastructural capacity of the country (e.g., through the use of questionnaires, the establishment of patient-reported outcome measures [PROMs] and patient-reported experience measures [PREMs] or the inclusion of patients in its formal channels for breast cancer care and pharmaceutical-related decision making).
	C.1.7	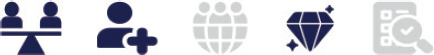	Country has a national program, policy or framework to guarantee access to a personalised survivorship care plan for treated patients, which includes surveillance for BC recurrence (physical examination, symptoms assessment), assessment and management of physical and psychosocial long-term and late effects of breast cancer and its treatment and the provision of patient education on self-management and wellness.
	**C.2**		Ensure treatment completion for more than 80% of breast cancer patients.
	C.2.1	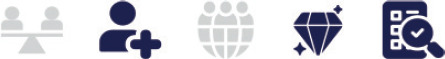	Proportion of patients who complete their recommended treatment out of the total number of patients treated.
D. Strong and resilient healthcare systems Strengthen overall health system capacity for health promotion, and breast cancer diagnosis, treatment, and care	D.1		Ensure and strengthen the quality of essential healthcare systems regarding infrastructure, capability, capacity, knowledge, and resources.
	D.1.1	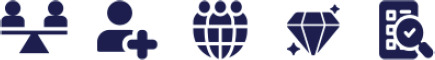	Country has identified and established sustainable sources of funding dedicated to subsidising expanded BC patient access and addressing breast-cancer focused programs and activities, even when included under the scope of broader strategies, plans and initiatives (e.g., prevention, non-communicable disease, women's health and health literacy).
	D.1.2	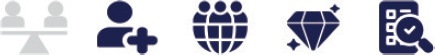	Number of BC-specialised healthcare professionals (medical oncologists, radiologists, radiation oncologists, pathologists, surgical oncologists, oncology pharmacists, general practitioners, nurses, psychologists, palliative care specialists and geneticists) per 10,000 cancer patients.
	D.1.3	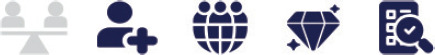	Number of specialised hospital units or departments that provide multidisciplinary BC care per 10,000 cancer patients.
	**D.2**		Improve availability of data regarding BC epidemiology, as well as availability, access, quality, and impact of breast cancer care and management services and programs.
	D.2.1	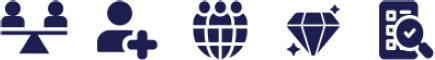	Country collects population-wide data through national or regional cancer registries, which cover BC and include comprehensive information regarding patient characteristics and disease stage.
	D.2.2	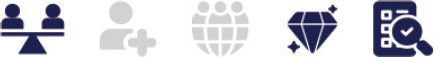	Yearly breast cancer mortality and/or 5-year survival, where available.
	**D.3**		Ensure availability of quality BC services and continuum of care, through availability and application of evidence-based, comprehensive clinical practice guidelines and optimal coordination of care and patient navigation across levels.
	D.3.1	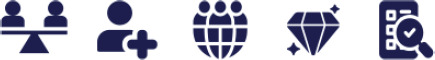	Country has established a framework to promote and monitor the adoption, dissemination and adherence of resource-appropriate guidance based on evidence-based clinical practice guidelines for the prevention, diagnosis, treatment, follow-up and survivorship care of BC.
	D.3.2	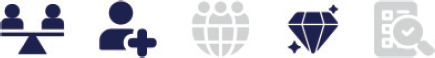	Country has established well-defined service integration/patient navigation mechanisms to facilitate access to integrated and coordinated BC care services across the continuum of care.
	D.3.3	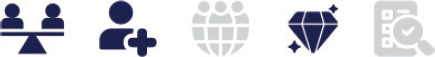	Country has established a framework to ensure patient engagement through platforms for participation in healthcare decision making and health service planning and design, e.g., through patient advisory standing committees or systematic open consultation with patient groups.

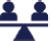
 Health equity 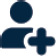 Patient centricity 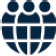 Universal access to health 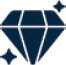 Healthcare quality 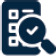 Treatment effectiveness

## Supplementary Material

**Table S1.** The Breast Cancer Care Quality Index (BCCQI) – description of targets.

Target	Rationale & Purpose
Measurable metrics with defined timeframes, articulated through clear statements or quantitative benchmarks.	The justification for assessing each target
**A. Early breast cancer detection**	
**A1** Establish appropriate national programs, policies or frameworks to ensure availability of and equitable access to affordable and appropriate breast cancer early detection programs and services.	This target references programs, policies and frameworks to ensure inclusivity and avoid being overly prescriptive or ambitious. This approach acknowledges that countries have varying capacities and may not be able to implement comprehensive policies or frameworks from the outset. Instead, it allows flexibility for countries to initially establish programs, which can be supported or developed in collaboration with external partners and donors as needed. Aligned with international best practices, the target highlights the need for ensuring access to BC early detection programs and services, with no financial barriers which might deter prompt access by individuals, in close proximity to the population, including the primary health care/community level in which individuals with suspicious findings are identified and referred for diagnostic work-up, as recommended by the GBCI framework [1]. This is also aligned to the WHO Noncommunicable disease facility-based monitoring guidance, which recommends that primary health care/community facilities referrals for mammography screening to facilities with capacity for diagnosis [2]. The target seeks to address the lack of awareness among the population and healthcare workers by promoting the inclusion of awareness and education programs as a key component of national health-related frameworks to ensure that both groups are well-informed about early detection and BC [3]. The target highlights the importance of equitable and affordable BC early detection programs and services to ensure that the benefits of early diagnosis are accessible to all individuals, with no group being left behind [3]. Dedicated policies, frameworks or programs would represent a foundational step to recognise the importance of BC early detection programs and services and set up the country to implement them.
**A2** Ensure availability of and access to affordable and appropriate breast cancer early detection programs and services.	To meet this target, countries must establish and deliver BC early detection programs and services at the primary health care and community levels, ensuring accessibility for the population [1]. These initiatives highlight BC awareness, education on early signs and symptoms, BC self-examinations and resource-appropriate screening programs for women with elevated risk for BC. Progress toward this target can be measured by the proportion of women with elevated risk screened within a specified period. According to international guidelines, risk evaluation must consider factors such as age, family history, personal history or genetic predisposition [4]. However, countries may utilise more comprehensive risk assessment approaches, when possible, based on the specific context and situation. In addition, countries should maximise the use of their resources by prioritising culturally sensitive, resource-appropriate and cost-effective strategies designed in collaboration with local authorities. This target encourages countries to develop effective and sustainable screening strategies that are aligned with current healthcare infrastructure and available resources, while ensuring access for those most in need.
**A3** Ensure that at least 60% of invasive breast cancers are diagnosed at stage I or II.	The key performance indicator for pillar 1 from the GBCI framework aims for at least 60% of invasive breast cancers are stage I or II at diagnosis. This is based on data indicating that every country experiencing a sustained annual decline of 2% or more in BC mortality rates for at least three consecutive years has achieved this level of early detection [1, 5]. For assessing this target, the indicator used is the proportion of invasive cancers diagnosed at stage I or II according to TNM anatomic and/or pathological staging, given that TNM remains the most widely adopted cancer staging system [6]. Anatomic and pathological staging offer critical insights into tumour size, nodal involvement and metastasis [7–9], enabling an assessment of disease progression and the success of BC early detection programs and services.
**B. Timely breast cancer diagnosis**	
**B1** Ensure timely and equitable access to affordable and quality diagnostic services.	Countries should develop a national policy or framework to guarantee timely and equitable access to affordable and quality diagnostic services that enable accurate pathological confirmation and prompt intervention. This target represents a critical milestone in ensuring that the country establishes specialised diagnostic services, delivered uniformly across the country [1, 3].
**B2** Ensure diagnosis completion (including clinical assessment, imaging, tissue sampling, pathological analysis, HR/HER2 testing) within 2 months from first access/presentation due to a suspicious finding.	The key performance indicator for pillar 2 from the GBCI framework aims that BC is diagnosed within 2 months from initial presentation due to a suspicious finding. BC early clinical detection can improve outcomes only if it is followed by a timely pathological diagnosis and the initiation of high-quality treatment [1]. According to the timeframe outlined in the GBCI framework and by international guidelines, treatment should commence within 3 months of the initial presentation, as studies show that delays beyond the period of 3 months lead to lower rates of BC survival [1, 10]. To meet this goal, achieving a definitive diagnosis within 2 months is critical, setting the stage for initiating treatment within 3 months. The diagnostic process includes clinical evaluation, imaging, tissue sampling, pathological analysis, HR/HER2 testing and germline genetic testing when indicated and available.^3^ After a cancer diagnosis is confirmed, staging is also needed to assess the size of the tumour, its location and whether the cancer has spread to nearby lymph nodes or other parts of the body, thus guiding treatment [1].
**C. Comprehensive breast cancer management**	
**C1** Ensure timely, equitable, and affordable access to quality, comprehensive, and multidisciplinary breast cancer care and management, including supportive services like pain management, physiotherapy, supportive medications, lymphedema management, psycho-oncology and oncofertility.	The establishment of a national policy or framework to guarantee timely, equitable and affordable access to comprehensive multidisciplinary care, from treatment initiation to completion is needed, as limited access to services, high out-of-pocket costs and lack of consideration for the patient perspective contribute to treatment interruptions or abandonment among patients, and these gaps in care can result in poorer outcomes, such as disease recurrence and mortality [3]. The timeliness of treatment initiation is relevant as, by establishing a reasonable and achievable threshold, this target aims to maximise patient benefits while minimising the adverse effects of delays, particularly for more aggressive cancers [10, 11]. The GBCI framework, international guidelines and relevant studies recommend that treatment commence within 3 months from first access/presentation due to a suspicious finding [1, 10]. The appropriateness of care, through the delivery of a specific treatment approach for each subtype of BC, is outlined by international guidelines. During or after treatment, supportive services are crucial to enhance quality of life and address the psychological and emotional impact of BC. These include pain management, physiotherapy, supportive medications, lymphedema management, psycho-oncology and oncofertility [1, 12]. Surveillance for BC recurrence, assessment and management of long-term and late effects of BC and its treatment, patient education on self-management and wellness, are essential to optimise outcomes and quality of life after recovery. These are the critical components typically included in a survivorship cancer care plan, which is a critical tool that countries should develop and adopt to facilitate women’s transition back into a socially active and productive life [13, 14]. LMICs can also rely on specific guidance available for the development and implementation of survivorship care plans in resource-constraint settings [15]. Patient-based metrics such as PREMs and PROMs are also increasingly being used to improve care delivery and are becoming part of routine clinical practice [16], mostly in high-income countries [17]. Even though this is not common yet in other world regions, there are various ways, with differing levels of technological complexity, to incorporate patient perspectives into care [18–21], which is important given that the lack of consideration for patient perspective affects treatment success and the patient psychological and emotional status [22, 23].
**C2** Ensure treatment completion for more than 80% of breast cancer patients.	The key performance indicator for pillar 1 from the GBCI framework aims for over 80% of BC patients to complete their recommended treatment. This target is based on the principle that treatment completion is a critical factor influencing BC patient outcomes [24]. Treatment completion refers to the successful fulfilment of all steps in the therapeutic sequence, except in cases where treatment is discontinued for clinical reasons (e.g., excessive toxicity) or when its potential benefits are outweighed by its risks [1, 25].
**D. Strong and resilient healthcare systems**	
**D1** Ensure and strengthen the quality of essential healthcare systems regarding infrastructure, capability, capacity, knowledge, and resources.	The quality of healthcare systems regarding infrastructure, capability, capacity, knowledge and resources must be strengthened, as these will ultimately constitute the backbone on which the provision of BC services will rely upon. Healthcare systems must be supported by reliable and sustainable sources of funding dedicated to subsidising expanded patient access to appropriate BC detection, diagnostic and management programs and services [26]. Specific funds should also be allocated to cover BC-focused programs and activities, even when included under the scope of broader strategies, plans and initiatives, such as prevention, non-communicable disease, women's health and health literacy. Specialised hospital units or departments should be available to provide multidisciplinary BC care. This is based on evidence showing that the availability of public hospitals is associated with lower BC mortality rates [5]. Comprehensive BC care requires the involvement of various specialised healthcare professionals, such as physicians, nurses, pharmacists, technologists and psychologists [3]. Among physicians, this includes medical oncologists, radiologists, radiation oncologists, pathologists, surgical oncologists, general practitioners, palliative care specialists and geneticists [1, 2, 27, 28].
**D2** Improve availability of data regarding breast cancer epidemiology, as well as availability, access, quality and impact of breast cancer care and management services and programs.	This target is assessed through the analysis of the existence of a cancer registry, either national or regional, that covers BC and includes information regarding patient characteristics and disease stage. These statistics in BC in a defined population provide a framework for assessing and controlling the impact of cancer in the community [29]. Population-based cancer registries have been in operation in many countries for decades, adhering to international standards set by the International Agency for Research on Cancer (IARC) [29]. However, generally speaking, the coverage of cancer registries is limited [30] and countries rarely systematically record BC stage at diagnosis or track relapses due to challenges in long-term patient follow-up. Nevertheless, complete data reporting is essential and should include information regarding patient demographics, disease stage and, ideally, other relevant disease-related characteristics. The target is also assessed using the outcome indicator of yearly BC mortality and/or 5-year survival, where available, as these metrics are commonly used to determine the effectiveness of interventions, the overall quality of care and inter-country comparisons [17, 31, 32]. The goal is to begin by recording either mortality or survival – whichever is more feasible – and gradually expand to comprehensive reporting on both mortality and survival across all countries as capacity increases.
**D3** Ensure availability of quality breast cancer services and continuum of care, through availability and application of evidence-based, comprehensive clinical practice guidelines and optimal coordination of care and patient navigation across levels.	This target is assessed by the establishment of a framework to promote, monitor and support the use of resource-adequate, evidence-based CPGs for BC prevention, diagnosis, treatment, follow-up and survivorship care, because evidence shows that such guidelines can enhance care quality and improve patient outcomes [33]. Organised navigation across care levels is essential to address breast health needs promptly and effectively [1]. In its technical brief *Patient navigation for early detection, diagnosis and treatment of BC*, the WHO emphasises how patient navigation helps address patient- and healthcare system-barriers to timely BC diagnosis and treatment. More importantly, it facilitates equitable access to quality care, especially for marginalised and vulnerable populations. The brief also provides guidance to support Member States in the establishment of BC navigation programs. Considering this evidence and the WHO’s work, this target also assesses the establishment of a framework that ensures patient engagement is fostered through platforms that enable their participation in healthcare decision making [1].

**Table S2.** The Breast Cancer Care Quality Index (BCCQI) – description of indicators.

Indicators	Rationale & purpose	Additional considerations	Data source(s)	Method of calculation	Type of indicator
Key indicators of the BCCQI that translate targets into measurable metrics	The justification for assessing each indicator and intended use	Additional factors to consider for the effective implementation of the indicator	The source(s) from which data and information should be collected	Process or formula used to calculate quantitative indicators	Type of the indicator according to Donabedian's quality of care model (Structure, Process, Outcome)
**Early detection Promote early detection of BC**					
**A1.1** Country has a national program, policy or framework that sets out appropriate measures to ensure availability of and equitable access to affordable and appropriate primary health care or community BC early detection programs and services, where individuals with suspicious findings are identified and referred for diagnostic work-up. *Domains advanced: Health equity, patient centricity, universal access to health, effectiveness of treatment*	The availability of and equitable access to affordable, appropriate BC early detection programs and services, delivered through primary health care and community services, facilitates early detection of BC. This indicator seeks to assess whether a country has established a national program, policy or framework that outlines appropriate measures to ensure BC early detection programs and services in which patients with suspicious findings are identified and referred for diagnostic work-up.	The country has a national program, policy or framework that establishes resource-appropriate BC early detection programs and services to identify individuals with suspicious signs and symptoms and refer them to more specialised cancer diagnostic services.	**National government policies** (e.g., laws, decrees and executive orders): national health laws; access to medicines laws; and presidential decrees. **Ministry-level policies** (e.g. Ministry of Health): health regulations; ministerial resolutions; and care protocols. **National health plans and programs** (national plans, programs, schemes and frameworks): e.g., strategic and disease-specific plans and programs; financing schemes; and surveillance frameworks. **Subnational regulations/policies** (state, province, regional and local regulations, policies and care protocols): e.g., public health and safety regulations; healthcare facility and professional regulations; health financing policies; pharmaceutical and drug control regulations; care protocols; and health data protection and privacy laws.	Not applicable	Structure
**A1.2** Breast cancer awareness and education programs for the public and healthcare workers to support early detection and increase recognition of early signs and/or symptoms of BC are included in national health-related frameworks.	Enhancing understanding of BC risk factors, signs and symptoms among the public and healthcare professionals is critical to improve the rate of BC detected at stage I and II.	The country has cancer awareness and education programs for the public and healthcare workers included in national health-related frameworks.	**National government policies** (e.g., laws, decrees, executive orders): national health laws; access to medicines laws and presidential decrees. **Ministry-level policies** (e.g., Ministry of Health): health regulations; ministerial resolutions and care protocols.	Not applicable	Structure
*Domains advanced: Health equity, patient centricity, effectiveness of treatment*	This indicator seeks to assess whether appropriate awareness and education-raising programs for the public and healthcare workers have been included in national health-related frameworks.	To fulfill this indicator, awareness and education programs can be included in different types of national health-related frameworks (e.g., national health plan, national plan for prevention and control of NCDs or cancer); however, these should focus specifically on improving knowledge about BC, its early signs and/or symptoms, as well as, whenever possible, treatment options and prognosis. The programs should address the public and healthcare workers, and can involve the participation of patient advocates, family members, peers, medical professionals, media, academic teachers and cultural and community leaders.	**National health plans and programs** (national plans, programs, schemes and frameworks): e.g., strategic and disease-specific plans and programs; financing schemes; and surveillance frameworks. **Subnational regulations/policies** (state, province, regional and local regulations, policies and care protocols): e.g., public health and safety regulations; healthcare facility and professional regulations; health financing policies; pharmaceutical and drug control regulations; care protocols; and health data protection and privacy laws.		
**A2.1** Country establishes and guarantees the execution of affordable and appropriate primary health care and community BC early detection programs and services for the identification and referral of individuals with suspicious findings for diagnostic work-up. *Domains advanced: Health equity, patient centricity, universal access to health, effectiveness of treatment*	Prompt identification and referral of individuals with suspicious findings for diagnostic work-up promotes early diagnosis, potentially accelerating treatment initiation. This indicator seeks to assess whether appropriate BC early detection programs and services are executed.	The country ensures that BC early detection programs and services at the primary health care and community levels are executed. This indicator requires countries to design and provide affordable and accessible BC early detection programs and services, aligned with available resources and healthcare system capacity, to ensure that individuals with suspicious findings upon clinical examination are promptly identified and referred for diagnostic work-up.	**National government policies** (e.g., laws, decrees, executive orders): national health laws; access to medicines laws and presidential decrees. **Ministry-level policies** (e.g., Ministry of Health): health regulations; ministerial resolutions and care protocols. **National health plans and programs** (national plans, programs, schemes and frameworks): e.g., strategic and disease-specific plans and programs; financing schemes; and surveillance frameworks. **Subnational regulations/policies** (state, province, regional and local regulations, policies and care protocols): e.g., public health and safety regulations; healthcare facility and professional regulations; health financing policies; pharmaceutical and drug control regulations; care protocols; and health data protection and privacy laws. **Healthcare service delivery data:** data on healthcare service utilisation and medical records.	Not applicable	Structure
**A2.2 Proportion** of women at elevated risk of BC screened at least once every 2 years. *Domains advanced: Health equity, universal access to health, healthcare quality*	Screening programs aim to enhance early BC detection by routinely testing asymptomatic individuals, identifying the disease before noticeable signs or symptoms appear. This indicator seeks to enable assessment of progress in the screening of women at elevated risk of BC.	A BC screening program is an early detection approach in which women without symptoms are regularly invited to undergo tests to detect BC before symptoms appear. This indicator requires countries to monitor the proportion of women at elevated risk of BC who are screened. To monitor this indicator, countries should identify the population at elevated risk and assess the proportion of women within this group who are screened at least once every 2 years. To allow for local adaptation and acknowledgement that some countries may use more comprehensive risk assessment approaches, this indicator focuses on women at elevated risk of BC rather than strictly on the population at high risk. A screening frequency of at least once every 2 years aligns with international guidelines while allowing for more frequent screening, when feasible, to improve early detection rates in women at elevated risk of more aggressive forms of BC.	**Healthcare service delivery data:** data on healthcare service utilisation and medical records. **Demographic data:** population statistics and census data.	*Numerator:* • Number of women at elevated risk of BC screened *Denominator:* • Total number of women at elevated risk of BC	Process
**A3.1** Proportion of invasive cancers diagnosed at stage I or II according to TNM anatomic and/or pathological staging. *Domains advanced: Health equity, healthcare quality, effectiveness of treatment*	Countries with a consistent decline of 2% or more in BC mortality for at least 3 years have 60% or more of invasive BCs diagnosed at stages I or II. Conversely, no country with lower BC early detection rates has seen a sustained decline in mortality.	Proportion of invasive cancers diagnosed at stage I or II according to TNM anatomic and/or pathological staging. The tumour, node, metastasis (TNM) system is a staging tool used to classify the extent of BC according to primary tumour, regional lymph nodes and distant metastasis information. The staging classification helps guide treatment decisions.	**Healthcare service delivery data:** data on healthcare service utilisation and medical records. **Cancer registry**: stage at diagnosis	*Numerator:* • Number of invasive BC diagnosed at stage I or II according to TNM anatomic and/or pathological staging	Outcome
	This indicator seeks to enable assessment of progress towards earlier stage detection.	While an initial assessment of the TNM stage of the cancer is usually done during the initial clinical examination (anatomic TNM staging), cancer stage is often revised after the final pathology report provides a better assessment of the involvement of the lymph nodes (pathological staging). Countries should aim to achieve the GBCI-set target of at least 60% of invasive BC stage I or II at diagnosis for this indicator.		*Denominator:* • Total number of invasive BC diagnosed according to TNM anatomic and/or pathological staging	
**Timely diagnosis Ensure timely access to appropriate BC diagnosis**					
**B1.1** Country has a national policy or framework that guarantees prompt access to equitable, accessible, affordable and quality diagnostic services in specialised settings after a suspicious finding. *Domains advanced: Health equity, patient centricity, universal access to health, healthcare quality, effectiveness of treatment*	Timely completion of the necessary diagnostic work-up after a suspicious finding is crucial for diagnosis confirmation, cancer characteristic determination and appropriate treatment planning. This indicator seeks to ensure that countries develop and adopt a specific policy or framework applicable across all jurisdictions to guarantee the availability of specialised services for complete diagnosis, accessible to every woman with a suspicious finding upon breast clinical evaluation or initial screening.	The country has a policy or framework in place to ensure the availability and accessibility of specialised services for complete diagnosis after a suspicious finding. Essential requirements for diagnostic services must be defined by the framework, which must apply across all jurisdictions and strive for equitability, accessibility and affordability. By leveraging resource-stratified guidelines, countries can align BC diagnostic services to available resources and healthcare system capacity.	**National government policies** (e.g., laws, decrees and executive orders): national health laws, access to medicines laws and presidential decrees. **Ministry-level policies (**e.g., Ministry of Health): health regulations; ministerial resolutions and care protocols. **National health plans and programs** (national plans, programs, schemes and frameworks): e.g., strategic and disease-specific plans and programs; financing schemes; and surveillance frameworks. **Subnational regulations/policies** (state, province, regional and local regulations, policies and care protocols): e.g., public health and safety regulations; healthcare facility and professional regulations; health financing policies; pharmaceutical and drug control regulations; care protocols; and health data protection and privacy laws.	Not applicable	Structure
**B2.1** Proportion of patients with complete and appropriate diagnosis and staging (including clinical evaluation, imaging, tissue sampling, pathological analysis, HR/HER2 testing and germline genetic testing when indicated and available) within 2 months from first access/presentation due to a suspicious finding. *Domains advanced: Health equity, universal access to health, healthcare quality, effectiveness of treatment*	Early detection of BC can improve patient outcomes only if a timely, complete and accurate diagnosis facilitates the prompt initiation of the most effective treatment. This indicator seeks to enable assessment of progress towards timely completion of full BC diagnosis.	Proportion of complete diagnosis delivered within 2 months from first access/presentation. Diagnosis includes clinical evaluation, imaging, tissue sampling, pathological analysis, HR/HER2 testing and germline genetic testing when indicated. While desirable, genetic testing use might vary based on availability and patient preferences. Upon confirmation of cancer, staging helps determine the disease extent to guide treatment decision. Initial staging occurs during the diagnostic evaluation, but definitive staging might require tests and services available only at tertiary care level. Guidelines recommend that treatment should begin within 3 months of a patient’s first access/presentation due to a suspicious finding. A complete diagnosis within 2 months ensures at least 1 month for treatment initiation. If necessary, staging can be completed within this period or later, provided treatment is not delayed. Whenever possible, earlier treatment initiation is desirable to improve outcomes, particularly for BC patients with a poorer prognosis. The GBCI assesses whether diagnostic evaluation, imaging, tissue sampling and pathology are completed within 60 days as a key performance indicator for timely BC diagnosis.	**Healthcare service delivery data:** data on healthcare service utilisation, medical records. **Cancer registry**: Stage at diagnosis	*Numerator:* • Number of patients with a complete diagnosis within 2 months from first access/ presentation due to a suspicious finding *Denominator:* • Total number of patients with a suspicious finding who did not receive a complete diagnosis within 2 months from first access/ presentation	Outcome
**Comprehensive management Guarantee timely access to comprehensive BC treatment and care for all patients at all stages**					
**C1.1** Country has a national policy or framework to guarantee timely, equitable and affordable access to comprehensive multidisciplinary care, from treatment initiation to completion. *Domains advanced: Health equity, patient centricity, universal access to health, healthcare quality, effectiveness of treatment*	Delivery of high-quality multidisciplinary care, from treatment initiation to completion, is crucial to improve patient outcomes, quality of life and maximise survival rates of BC patients. This indicator seeks to ensure that countries adopt a specific policy or framework to guarantee access to comprehensive multidisciplinary care for all BC patients.	To establish or enhance BC treatment, this indicator requires countries to define the essential requirements for multidisciplinary care through a specific policy or framework applicable nationwide, across all jurisdictions. This must ensure that BC multidisciplinary care is provided in a timely, equitable and affordable manner, from treatment initiation to completion. By leveraging resource-stratified guidelines, countries can align the design of their BC multidisciplinary care services to available resources and healthcare system capacity.	**National government policies** (e.g., laws, decrees, executive orders): national health laws; access to medicines laws and presidential decrees. **Ministry-level policies (**e.g., Ministry of Health): health regulations; ministerial resolutions and care protocols. **National health plans and programs** (national plans, programs, schemes and frameworks): e.g., strategic and disease-specific plans and programs; financing schemes; and surveillance frameworks. **Subnational regulations/policies** (state, province, regional and local regulations, policies and care protocols): e.g., public health and safety regulations; healthcare facility and professional regulations; health financing policies; pharmaceutical and drug control regulations; care protocols; and health data protection and privacy laws.	Not applicable	Structure
**C1.2** Proportion of patients with confirmed diagnosis of BC who have their adequate multidisciplinary treatment initiated within 3 months from first access/presentation due to a suspicious finding. *Domains advanced: Health equity, patient centricity, healthcare quality*	Maximum effectiveness of BC treatment depends on prompt initiation of treatment. This indicator seeks to enable assessment of progress towards timely initiation of treatment for all patients.	Proportion of patients who had their treatment initiated within 3 months from first access/presentation due to a suspicious finding. Evidence-based guidelines recommend that treatment should start within 3 months from the first access/presentation due to a suspicious finding since studies show that delays beyond this period lead to lower rates of BC survival. Whenever possible, earlier treatment initiation is desirable to improve outcomes, particularly for BC patients with a poorer prognosis.	**Healthcare service delivery data:** Data on healthcare service utilisation and medical records. **Cancer registry**: diagnosis; and treatment information.	*Numerator:* • Number of patients who have their adequate multidisciplinary treatment initiated within 3 months from first access/ presentation due to a suspicious finding *Denominator:* • Total number of patients with a suspicious finding that did not have multidisciplinary treatment initiated within 3 months from first access/ presentation	Process
**C1.3** Proportion of triple-negative BC, HER2+ and HR+/HER- early BC patients that receive neoadjuvant therapy or primary surgery as their first treatment, in alignment with what is recommended by international guidelines. *Domains advanced: Patient centricity, healthcare quality, effectiveness of treatment*	International early BC guidelines outline the most effective treatments and interventions for each patient based on the specific BC type, stage and characteristics. Adhering to international guidelines’ recommendations is essential to maximising benefits, improving outcomes and enhancing survival rates for each patient. This indicator is a composite indicator that seeks to enable assessment of progress in ensuring that every early BC patient receives the most effective treatment and intervention regimen based on the specific BC type, stage and characteristics.	To evaluate this indicator, countries should independently track the proportion of patients receiving either neoadjuvant therapy or primary surgery as their initial treatment, in accordance with international guidelines. This assessment should be conducted across the following three patient subpopulations: • Triple-negative breast cancer (TNBC) • HER2-positive (HER2+) BC • Hormone receptor-positive, HER2-negative (HR+/HER-) BC This indicator seeks to enable countries to assess and monitor over time the appropriateness of BC care received by triple-negative BC, HER2+ and HR+/HER- patients, to identify critical gaps. **Note:** For international benchmarking of this indicator, it is necessary to consider that this proportion will vary depending on the spectrum of presenting stages.	**Healthcare service delivery data:** data on healthcare service utilisation and medical records. **Cancer registry**: Treatment information. **National regulatory authority:** Decisions; regulations; guidance; formularies and lists of reimbursed and approved medicines and health technologies.	*Numerator:* • Number of BC patients in each subpopulation (**TNBC, HER2+, HR+/HER-**) who receive either: • **Neoadjuvant therapy** when recommended by international guidelines, OR • **Primary surgery** when recommended by international guidelines *Denominator:* • Total number of newly diagnosed breast cancer patients in each subpopulation (**TNBC, HER2+, HR+/HER-**) who are eligible for either neoadjuvant therapy or primary surgery as their first treatment.	Process
**C1.4** Proportion of patients with hormone receptor-positive invasive breast cancer who received endocrine therapy (ET) in alignment with international guidelines. *Domains advanced: Patient centricity, healthcare quality, effectiveness of treatment*	According to ESMO guidance, adjuvant ET ‘*is almost universal for patients with hormone receptor-positive invasive breast cancer of any stage and HER2 status*’. This is based on evidence that adjuvant ET ‘*reduces the risk of locoregional recurrence, distant metastatic recurrence and contralateral breast cancer, while improving overall survival (OS)*’.	Proportion of patients with hormone receptor-positive cancer who receive adjuvant ET in alignment with international guidelines.	**Healthcare service delivery data:** data on healthcare service utilisation; and medical records. **Cancer registry**: treatment information.	*Numerator:* • Number of patients with hormone receptor-positive invasive BC that receives adjuvant ET, in alignment with international guidelines *Denominator:* • Total number of patients with hormone receptor-positive invasive BC	Process
	This indicator seeks to enable assessment of progress in ensuring that hormone receptor-positive invasive BC receives the most effective treatment and intervention regimen in alignment with international guidelines.	For hormone receptor-positive BC, both surgery and neoadjuvant chemotherapy may be considered as initial treatment options based on individual recurrence risk and anatomical factors such as tumour size and nodal status. However, adjuvant ET is always considered for these patients due to evidence of its benefits on disease progression and overall survival.	**National regulatory authority:** decisions, regulations; guidance; formularies; and lists of reimbursed and approved medicines and health technologies.		
**C1.5** Proportion of patients that receive adequate supportive services and palliative care they need, e.g., pain management, physiotherapy, supportive medications, lymphedema management, psycho-oncology support, and oncofertility, out of total number of patients in treatment. *Domains advanced: Health equity, patient centricity, healthcare quality, effectiveness of treatment*	Supportive services, including pain management and palliative care, are an indispensable part of comprehensive BC management. They help enhance patient compliance, quality of life and outcomes, during treatment; and help manage psycho-emotional challenges in patients and survivors. This indicator seeks to enable assessment of progress in expanding the provision of supportive services and palliative care.	Proportion of patients who receive adequate supportive services and palliative care they need. Supportive services include a vast range of services, like for example pain management, physiotherapy, supportive medications, lymphedema management, psycho-oncology support and oncofertility. Palliative care approach serves as the cornerstone of patient-centered care, improving quality of life for cancer patients by managing highly debilitating symptoms such as nausea, fatigue, anxiety, delirium, confusion and depression throughout the disease course, especially in advanced stages. By leveraging resource-stratified guidelines, countries can adapt their supportive care services to available resources and healthcare system capability, while aiming to expand provision as capacity builds. The indicator must consider information collected as part of healthcare system performance assessment efforts. This information must integrate data from different sources, including healthcare professional surveys focusing on the number of patients obtaining access to the supportive services they would need.	**Healthcare system key performance indicator data or surveys:** information collected at the national level to assess the quality of services provided, developed through the analysis of available data on healthcare service provision and utilisation, medical records and specific surveys. **Healthcare service delivery data:** data on healthcare service utilisation; and medical records. **Cancer registry**: supportive services information.	*Numerator:* • Number of BC patients that receive supportive services and palliative care as indicated *Denominator:* • Total number of BC patients in treatment	Process
**C1.6** Country has provided guidance or is working towards the incorporation of patient perspective in its service quality assessment protocols for BC within the infrastructural capacity of the country (e.g., through the use of questionnaires, the establishment of patient-reported outcome measures [PROMs] and patient-reported experience measures [PREMs] or the inclusion of patients in its formal channels for BC care and pharmaceutical-related decision making). *Domains advanced: Health equity, patient centricity, healthcare quality, effectiveness of treatment*	Collection and analysis of patient-reported outcome measures (PROMs) has proven effective in assessing and reducing treatment-related toxicity. These measures along with patient-reported experience measures (PREMs) are crucial to ensure that choices made regarding BC treatment avoid detrimental impact on patients’ quality of life and drive improvements in BC care. This indicator seeks to ensure that the country is working toward the incorporation of patient perspective in its decision-making.	The country has incorporated – or is actively working toward incorporating – patient perspectives into its BC service quality assessment procedures. Patient perspectives can be integrated through various approaches, including the use of validated questionnaires for collecting patient-reported outcome measures (PROMs) and patient-reported experience measures (PREMs) or the inclusion of patients in formal decision-making processes related to BC healthcare and pharmaceuticals. PROMs and PREMs capture valuable insights into patients’ health status, treatment outcomes and overall healthcare experiences. To meet this indicator, the country should implement clear, nationwide guidance on the collection and analysis of PROMs and PREMs for BC service quality assessment. Additionally, launching pilot projects or establishing dedicated working groups would contribute to meeting this indicator. Alternatively, the country may establish systematic mechanisms for ensuring patient participation in formal BC healthcare and pharmaceutical policy discussions.	**National government policies** (e.g., laws, decrees, executive orders): national health laws; access to medicines laws; and presidential decrees. **Ministry-level policies (**e.g. Ministry of Health): health regulations; ministerial resolutions; and care protocols. **National health plans and programs** (national plans; programs; schemes and frameworks): e.g., strategic and disease-specific plans and programs, financing schemes and surveillance frameworks). **Subnational regulations/policies** (state, province, regional and local regulations, policies and care protocols): e.g., public health and safety regulations; healthcare facility and professional regulations; health financing policies; pharmaceutical and drug control regulations; care protocols; and health data protection and privacy laws.	Not applicable	Structure
**C1.7** Country has a national program, policy or framework to guarantee access to a personalised survivorship care plan for treated patients, which includes surveillance for BC recurrence (physical examination, symptoms assessment), assessment and management of physical and psychosocial long-term and late effects of BC and its treatment, and the provision of patient education on self-management and wellness. *Domains advanced: Health equity, patient centricity, healthcare quality*	Survivorship care is critical to address physical and psychosocial long-term and late effects of BC cancer and its treatment, and to prevent and detect recurrence and onset of new cancers after BC remission. This indicator seeks to ensure that the country has developed and adopted a program, policy or framework to ensure availability and access to adequate survivorship care.	The country has a national program, policy or framework to guarantee access to a personalised survivorship care plan for treated patients. Survivorship care transitions from active treatment to focusing on surveillance and health maintenance; it addresses the physical and psychosocial long-term and late effects of cancer and its treatment, promotes health maintaining behaviours including healthy lifestyle, self-management and wellness and includes appropriate surveillance to identify and assess recurrence. By leveraging guidance developed for design of survivorship care plans, countries can adapt their programs, plans and services to available resources and healthcare system capacity.	**National government policies** (e.g., laws, decrees, executive orders): national health laws; access to medicines laws; and presidential decrees. **Ministry-level policies (**e.g. Ministry of Health): health regulations; ministerial resolutions; and care protocols. **National Health Plans and Programs** (national plans, programs, schemes and frameworks): e.g., strategic and disease-specific plans and programs; financing schemes; and surveillance frameworks. **Subnational regulations/policies** (state, province, regional and local regulations, policies and care protocols): e.g., public health and safety regulations; healthcare facility and professional regulations; health financing policies; pharmaceutical and drug control regulations; care protocols; and health data protection and privacy laws.	Not applicable	Structure
**C2.1** Proportion of patients who complete their recommended treatment, out of the total number of patients treated. *Domains advanced: Patient centricity, healthcare quality, effectiveness of treatment*	Incomplete treatment leads to poorer patient outcomes, including recurrence and death, and also negatively affects quality of life. This indicator seeks to enable assessment of progress towards reduced treatment abandonment and improved compliance and adherence.	Proportion of patients who complete their recommended treatment. Completion of the recommended course of treatment without abandonment is associated with improved outcomes. To comply with the GBCI's target, in the long term, countries should aim to ensure that more than 80% of BC patients complete their recommended treatment. Cases in which treatment is discontinued for clinical reasons, such as excessive toxicity or when the projected benefit of the treatment is outweighed by its limitations, would not qualify as treatment abandonment but rather revision of the initially prescribed treatment regimen.	**Healthcare service delivery data:** Data on healthcare service utilisation; and medical records. **Cancer registry**: diagnosis and treatment information	*Numerator:* • Number of patients who complete their recommended treatment. *Denominator:* • Total number of patients that start treatment following BC diagnosis confirmation	Outcome
**Strong and resilient health system Strengthen overall health system capacity for health promotion, and BC diagnosis, treatment, and care**					
**D1.1** Country has identified and established sustainable sources of funding dedicated to subsidising expanded BC patient access and addressing BC focused programs and activities, even when included under the scope of broader strategies, plans, and initiatives (e.g., prevention, non-communicable disease, women's health, health literacy). *Domains advanced: Health equity, patient centricity, universal access to health, healthcare quality, effectiveness of treatment*	Adequate and sustainable funding is needed to finance BC programs, activities, and services and improve equity in BC outcomes across and within countries. This indicator seeks to help assess countries’ progress towards the identification of sustainable sources of funding dedicated to subsidising BC programs, activities and services.	The country has identified and established sustainable funding sources dedicated to expanding access to BC services and supporting BC-focused programs and initiatives. Sustainable funding is essential to ensure the continuity of planned activities on an annual basis. Each activity and program – whether part of dedicated BC plans, frameworks or broader initiatives such as prevention, non-communicable diseases, women's health or health literacy – should have clearly identified and allocated funding sources.	**National government policies** (e.g., laws, decrees, executive orders): national health laws; access to medicines laws and presidential decrees. **Ministry-level policies (**e.g., Ministry of Health): health regulations; ministerial resolutions and care protocols. **National health plans and programs** (national plans, programs, schemes and frameworks): e.g., strategic and disease-specific plans and programs; financing schemes and surveillance frameworks. **Subnational regulations/policies** (state, province, regional and local regulations, policies and care protocols): e.g., public health and safety regulations; healthcare facility and professional regulations; health financing policies; pharmaceutical and drug control regulations; care protocols; and health data protection and privacy laws.	Not applicable	Structure
**D1.2** Number of breast cancer-specialised healthcare professionals (medical oncologists, radiologists, radiation oncologists, pathologists, surgical oncologists, oncology pharmacists, general practitioners, nurses, psychologists, palliative care specialists and geneticists) per 10,000 cancer patients.	Well-trained and sufficient healthcare workforce is necessary to deliver timely, comprehensive and high-quality care across the continuum of BC care. This indicator seeks to enable an assessment of the available specialised healthcare workforce to identify gaps which might hinder the country’s capacity to meet the needs of BC patients.	Number of cancer specialised healthcare professionals in proportion to the number of cancer patients. Key healthcare professional figures that are needed to provide multidisciplinary care for BC patients include specialised healthcare providers, namely oncologists, radiologists, radiation oncologists, pathologists, surgical oncologists, general practitioners, nurses, psychologists, palliative care specialists and geneticists.	**Healthcare service delivery data:** healthcare workforce data. **Cancer registry**: cancer prevalence information	• *Numerator:* • Number of BC specialised healthcare professionals: • Medical Oncologists, • Radiologists, • Radiation oncologists, • Pathologists • Surgical oncologists, • Oncology pharmacists • General practitioners • Nurses • Psychologists	Process
*Domains advanced: Patient centricity, universal access to health, healthcare quality, effectiveness of treatment*				• Palliative care specialists and • Geneticists *Denominator:* • Total number of BC patients	
**D1.3** Number of specialised hospital units or departments that provide multidisciplinary BC care per 10,000 cancer patients. *Domains advanced: Patient centricity, universal access to health, healthcare quality, effectiveness of treatment*	Availability of sufficient and well-equipped infrastructure and facilities is critical to providing access to high-quality BC services across the continuum of care. This indicator seeks to enable assessment of the available specialised infrastructure to identify gaps which might hinder the country’s capacity to meet the needs of BC patients.	Number of specialised hospital units or departments that provide multidisciplinary BC care in proportion to the number of cancer patients. Key infrastructure elements needed to provide multidisciplinary care for BC patients include diagnostic facilities such as imaging and pathology labs, treatment centers equipped for surgery, chemotherapy and radiation therapy, which can be found in hospital units or departments that provide multidisciplinary BC care. Since proportion of specialised hospital units or departments is a minimal requirement, ideally, countries should assess suitability of their infrastructure and facilities for MDT BC care in alignment with the standards provided by the WHO IAEA Guidance on Setting up Cancer Centers or through a suitable accreditation program.	**Healthcare service delivery data:** healthcare facility data. **Ministry of Health (MoH):** monitoring and evaluation platforms, databases and systems. **Healthcare system key performance indicator data or surveys:** information collected at the national level to assess the quality of services provided, developed through the analysis of available data on healthcare service provision and utilisation and medical records. **Cancer registry**: cancer prevalence information.	*Numerator:* • Number of specialised hospital units or departments that provide multidisciplinary BC care *Denominator:* • Total number of BC patients	Process
**D2.1** Country collects population-wide data through national or regional cancer registries, which cover BC and include comprehensive information regarding patient characteristics and disease stage.	This indicator seeks to ensure that country has a population-wide data recording system which systematically collects critical BC related data.	The country collects population-wide data through national or regional cancer registries. Data collection is only the first step as needs identification, appropriate resource allocation and service planning requires accurate recording, reporting and analysis of nationwide data. This is crucial to inform healthcare decision-making and drive continuous improvements in BC care.	**Cancer registries:** patient information and stage at diagnosis.	Not applicable	Structure
*Domains advanced: Health equity, patient centricity, universal access to health, healthcare quality, effectiveness of treatment*		Registries can be national or regional, provided that they comply with international recommendations. Considering the current gaps existing in population-wide data regarding patient characteristics and disease stage, this information is considered the minimum starting point countries should strive for. All countries are expected to aim for accurate data recording and reporting of this information. Additional information countries should ideally collect, also to support progress assessment in the implementation of the BCCQI, includes: i. Age at diagnosis, ii. Gender, iii. Ethnicity/race (whenever possible), iv. Geographic location, v. Stage at diagnosis, vi. Tumour subtype, vii. Family history, viii. Known molecular markers and mutation, ix. Type of all treatments, x. Date of all treatments, xi. Recurrence, xii. Information on recurrence (local, regional and/or distant) and xiii. Outcome.			
**D2.2** Yearly BC mortality and/or 5-year survival where available. *Domains advanced: Health equity, healthcare quality, effectiveness of treatment*	To monitor and evaluate the impact of national efforts to improve the quality of BC services, accurate recording and reporting of BC mortality and survival rates are essential.	Statistics on yearly BC mortality and/or 5-year survival, according to data availability. Countries have varying capacities for data collection, and this indicator allows flexibility in reporting either mortality or survival data, depending on feasibility. However, a gradual transition toward comprehensive reporting of both metrics is encouraged as capacity improves.	**Data:** data on healthcare service utilisation; and medical records. **Cancer registry**: health outcomes information. **Demographic data:** population statistics and census data.	Yearly BC mortality, indicated as cancer mortality (rate per 100 000 persons per year): *Numerator:* • Number of BC deaths in a given period X 100,000	Outcome
	This indicator enables the assessment of a country's progress in reducing BC mortality and improving patient survival.	Ideally, countries should adopt a standardised methodology for data tracking and reporting. Additionally, while 5-year overall survival is commonly reported for BC, 10-year data is also important, particularly for hormone receptor-positive cases, where recurrence may occur later.		*Denominator:* • Total population • 5-year survival, indicated as the percentage of people that survive 5 years after their diagnosis *Numerator:* • Number of BC patients alive 5 years of their diagnosis *Denominator:* • Number of BC patients diagnosed	
**D3.1** Country has established a framework to promote and monitor the adoption, dissemination, and adherence of resource-appropriate guidance based on evidence-based clinical practice guidelines for the prevention, diagnosis, treatment, follow-up and survivorship care of BC. *Domains advanced: Health equity, patient centricity, universal access to health, healthcare quality, effectiveness of treatment*	Evidence shows that evidence-based clinical practice guidelines can enhance the quality of BC care provided, ultimately improving patient outcomes. In addition, they can help identify research gaps and guide healthcare policy and resource allocation. This indicator seeks to ensure that countries establish a framework dedicated to guaranteeing adherence of resource-appropriate, evidence-based BC clinical practice guidelines.	The country has established a framework to promote and monitor adoption, dissemination and adherence to resource-stratified, evidence-based clinical practice guidance. This is crucial to ensure delivery of consistent, evidence-based, high-quality care, thereby improving patient outcomes, reducing variability in clinical practices and optimising use of healthcare resources. Resource-appropriate guidance based on international evidence-based, resource-stratified clinical practice guidelines should be developed by countries and address prevention, diagnosis, treatment, follow-up and survivorship care.	**National government policies** (e.g., laws, decrees, executive orders): national health laws; access to medicines laws; and presidential decrees. **Ministry-level policies (**e.g., Ministry of Health): health regulations; ministerial resolutions and care protocols. **National health plans and programs** (national plans, programs, schemes and frameworks): e.g., strategic and disease-specific plans and programs; financing schemes; and surveillance frameworks. **Subnational regulations/policies** (state, province, regional and local regulations, policies and care protocols): e.g., public health and safety regulations; healthcare facility and professional regulations; health financing policies; pharmaceutical and drug control regulations; care protocols; and health data protection and privacy laws.	Not applicable	Structure
**D3.2** Country has established well-defined service integration/patient navigation mechanisms to facilitate access to integrated and coordinated BC care services across the continuum of care. *Domains advanced: Health equity, patient centricity, healthcare quality*	Patient navigation has been shown to be effective in helping address patient- and healthcare system- barriers to timely BC diagnosis and treatment, facilitating equitable access to quality care, especially for marginalised and vulnerable populations. This indicator seeks to assess whether the country established well-defined service integration/patient navigation mechanisms.	The country has established well-defined service integration/patient navigation mechanisms. Patient navigation mechanisms are critical for guiding individuals through the complex BC care pathway, from symptom recognition and early detection to diagnosis, treatment and follow-up care. These systems enhance care continuity by facilitating timely referrals and access to multidisciplinary, integrated and coordinated services, ensuring accurate diagnosis and comprehensive treatment. A well-defined service is documented and effectively disseminated throughout the healthcare system. Ideally, patient navigation systems should be monitored for effectiveness, ensuring that patients receive timely care, are supported throughout their treatment journey and experience minimal delays or fragmentation in care.	**National government policies** (e.g., laws, decrees, executive orders): national health laws; access to medicines laws; and presidential decrees. **Ministry-level policies (**e.g., Ministry of Health): health regulations; ministerial resolutions; and care protocols. **National Health Plans and Programs** (national plans, programs, schemes and frameworks): e.g., strategic and disease-specific plans and programs; financing schemes and surveillance frameworks. **Subnational regulations/policies** (state, province, regional and local regulations, policies and care protocols): e.g., public health and safety regulations; healthcare facility and professional regulations; health financing policies; pharmaceutical and drug control regulations; care protocols; and health data protection and privacy laws.	Not applicable	Structure
**D3.3** Country has established a framework to ensure patient engagement through platforms for participation in healthcare decision making and health service planning and design, e.g., through patient advisory standing committees or systematic open consultation with patient groups.	Patient engagement is crucial for ensuring that healthcare services and policies are patient-centered and address the real needs and preferences of BC patients.	The country has established a framework to ensure patient engagement through platforms for participation in healthcare decision-making and health service planning and design.	**National government policies** (e.g., laws, decrees, executive orders): national health laws; access to medicines laws; and presidential decrees.	Not applicable	Structure
*Domains advanced: Health equity, patient centricity, healthcare quality*	This indicator seeks to assess whether the country has adopted a framework to establish formal channels for patient engagement in broad healthcare decision-making and health service planning and design.	Patient engagement can be achieved through various platforms, for example: • Patient advisory standing committees, which provide a formal channel for patients to share their insights and experiences. • Systematic open consultations with patient groups, which involve gathering feedback on their needs and experiences in order to inform healthcare planning and decision making. Ideally, these platforms should be formalised and institutionalised to ensure the systematic integration of patient perspectives into policy development and service enhancement. Institutionalising these processes guarantees a structured approach to incorporating patient input in decision making. Additionally, these platforms should include transparent mechanisms for reporting patient contributions and their impact, fostering accountability and continuous improvement.	**Ministry-level policies (**e.g., Ministry of Health): health regulations, ministerial resolutions and care protocols. **National health plans and programs** (national plans, programs, schemes and frameworks): e.g., strategic and disease-specific plans and programs; financing schemes; and surveillance frameworks. **Subnational regulations/policies** (state, province, regional and local regulations, policies and care protocols): e.g., public health and safety regulations; healthcare facility and professional regulations; health financing policies; pharmaceutical and drug control regulations; care protocols; and health data protection and privacy laws.		
